# Genome-Wide Identification, Characterization and Expression Analysis of the *CIPK* Gene Family in Potato (*Solanum tuberosum* L.) and the Role of *StCIPK10* in Response to Drought and Osmotic Stress

**DOI:** 10.3390/ijms222413535

**Published:** 2021-12-16

**Authors:** Rui Ma, Weigang Liu, Shigui Li, Xi Zhu, Jiangwei Yang, Ning Zhang, Huaijun Si

**Affiliations:** 1 State Key Laboratory of Aridland Crop Science, Gansu Agricultural University, Lanzhou 730070, China; mr447233254@126.com (R.M.); wgliu@st.gsau.edu.cn (W.L.); lisg@st.gsau.edu.cn (S.L.); zx2020yx@163.com (X.Z.); yyangjiangwei@126.com (J.Y.); ningzh@gsau.edu.cn (N.Z.); 2 College of Agronomy, Gansu Agricultural University, Lanzhou 730070, China; 3 College of Life Science and Technology, Gansu Agricultural University, Lanzhou 730070, China; 4 Dingxi Academy of Agricultural Sciences, Dingxi 743000, China

**Keywords:** potato, genome-wide, *CIPK* gene family, gene expression, drought and osmotic tolerance, ABA

## Abstract

The potato (*Solanum tuberosum* L.), one of the most important food crops worldwide, is sensitive to environmental stresses. Sensor–responder complexes comprising calcineurin B-like (CBL) proteins and CBL-interacting protein kinases (CIPKs) not only modulate plant growth and development but also mediate numerous stress responses. Here, using a Hidden Markov Model and BLAST searches, 27 *CIPK* genes were identified in potato and divided into five groups by phylogenetic analysis and into two clades (intron-poor and intron-rich) by gene structure analysis. Quantitative reverse-transcription PCR (qRT-PCR) assays revealed that *StCIPK* genes play important roles in plant growth, development and abiotic stress tolerance. Up-regulated expression of *StCIPK10* was significantly induced by drought, PEG6000 and ABA. StCIPK10 enhances both the ability of potato to scavenge reactive oxygen species and the content of corresponding osmoregulation substances, thereby strengthening tolerance to drought and osmotic stress. StCIPK10 is located at the intersection between the abscisic acid and abiotic stress signaling pathways, which control both root growth and stomatal closure in potato. In addition, StCIPK10 interacts with StCBL1, StCBL4, StCBL6, StCBL7, StCBL8, StCBL11 and StCBL12, and is specifically recruited to the plasma membrane by StCBL11.

## 1. Introduction

During their evolution, plants have developed a set of complete signal transduction systems to adapt to biotic and abiotic stresses [1]. As a secondary messenger, calcium (Ca^2+^) integrates a variety of signals and is involved in many biochemical reactions in plants [2]. The Ca^2+^ signal is generated by variation in the intracellular transient Ca^2+^ concentration, which is perceived and decoded by various sensors, including calmodulin (CaM), calcineurin B-like (CBL) proteins, calmodulin-like (CML) proteins and Ca^2+^-dependent protein kinases (CDPKs) [3,4]. After receiving the Ca^2+^ signal, these sensors not only activate downstream components via phosphorylation but also interact with these downstream components, triggering a range of physiological and biochemical reactions [5].

CBL proteins lack a kinase domain, so they must bind to CBL-interacting protein kinases (CIPKs) specifically in order to function [6]. Following the identification of the CBL family, CIPKs were identified as a new family of protein kinases by high-throughput screening of a cDNA library of Arabidopsis (*Arabidopsis thaliana*) [7]. CIPKs are a group of plant-specific serine/threonine (Ser/Thr) kinases belonging to the SnRK3 protein family and play an important role in signal transduction via the CBL–CIPK module [1]. The core region of CIPKs includes an *N*-terminal catalytic domain and a *C*-terminal regulatory domain [8]. The *N*-terminal catalytic domain contains an ATP-binding loop and an activation segment, similar to the yeast SNF1 kinases in protein structure [7]. The *C*-terminal regulatory domain contains a highly conserved NAF–FISL motif, which mediates interactions between CBL proteins and CIPKs [9]. The binding of free CBL protein to Ca^2+^ exposes its hydrophobic surface, which interacts with the NAF–FISL motif of CIPK, thus forming a CBL–CIPK module [10]. The structure of the CBL–CIPK module is stabilized by hydrogen bonds and hydrophobic interactions [11]. Phosphorylation of CBL proteins is another proven regulatory mechanism that activates the function of the CBL–CIPK module. This phosphorylation, which has been discovered in almost all CBL–CIPK modules, enhances the interaction of the CBL protein with the CIPK [12]. The phosphorylation site of CBLs is a conserved Ser residue located in the *C*-terminal domain [13]. The CBL–CIPK module represents a plant-specific Ca^2+^ signal-decoding tool.

CIPKs play an important regulatory role in plant growth and development and abiotic stress tolerance. The CBL–CIPK module has been shown to improve plant stress tolerance by regulating the concentration of ions in plant cells [14]. In 1996, the Salt Overly Sensitive (SOS) signaling pathway was identified for the first time in Arabidopsis, and the core components of this pathway were identified as AtCBL4 (SOS3), AtCIPK24 (SOS2) and the Na^+^/H^+^ antiporter (SOS1) [15]. The AtCBL4–AtCIPK24 module phosphorylates and activates the Na^+^/H^+^ antiporter at the plasma membrane, which removes excess Na^+^ from the cell, thus improving salt tolerance [15]. AtCBL10–AtCIPK24 also interacts with the Na^+^/H^+^ exchanger (AtNHX) located on the tonoplast, which transports excess Na^+^ from the cytoplasm into the vacuole [16]. The AtCBL1/9–AtCIPK23 module promotes the absorption of potassium ions (K^+^) by phosphorylating a voltage-gated potassium transporter, Arabidopsis K^+^ transporter 1 (AKT1), under low K^+^ stress [17]. The absorption of K^+^ causes stomatal closure in leaves, thus improving drought tolerance [18]. The AtCBL1/9–AtCIPK1 module participates in the regulation of salt, drought and cold responses, while the AtCBL1/9-AtCIPK26 module participates in the regulation of reactive oxygen species (ROS) signaling in *Arabidopsis*. [19,20]. The AtCBL9–AtCIPK3 module is implicated in the regulation of Arabidopsis abscisic acid (ABA) signaling in seed germination [21]. Overexpression and mutation of *CIPK* genes alter the level of abiotic stress tolerance in plants. For example, overexpression of *AtCIPK6* increased salt tolerance in *Arabidopsis*, whereas mutation of *AtCIPK24* caused salt sensitivity [22]. Heterologous expression of maize *ZmCIPK16* in *Arabidopsis* enhanced salt tolerance [23]. In rice (*Oryza sativa* L.), cold stress up-regulates the *OsCIPK3* gene, and causes, in turn, the overexpression of *OsCIPK3* enhanced cold resistance [24]. Heterologous expression of wheat (*Triticum aestivum* L.) *TaCIPK2*, *TaCIPK23* or *TaCIPK27* in *Arabidopsis* improved the drought tolerance of transgenic plants through the ABA signaling pathway [25]. Overexpression of the apple (*Malus domestica* L.) *MdCIPK6L* gene in tomato (*Solanum lycopersicum* L.) enhanced drought and salt tolerance, possibly because MdCIPK6L is involved in the SOS pathway [26]. The CcCBL1–CcCIPK14 module regulated drought tolerance of pigeon pea (*Cajanus cajan*) by enhancing flavonoid biosynthesis [27]. These studies demonstrated that CIPK genes promote the adaptation of plants to adverse environmental conditions.

The *CIPK* gene family has been identified in many plants including *Arabidopsis*, wheat, eggplant (*Solanum melongena* L.), grapevine (*Vitis vinifera* L.), pepper (*Capsicum annuum* L.) and canola (*Brassica napus* L.) [28,29,30,31,32,33]. Potato (*Solanum tuberosum* L.) is one of the most nutrient-rich foods and most widely planted crops in the world [34]. However, yield and quality of potato are threatened by various abiotic stresses, especially drought and salt [35]. In particular, due to the shallow root system of potato, the depth of its rooting zone is between 50 cm and 80 cm, leading to drought stress, which has an extremely adverse effect on potato [36]. In addition to drought stress, potato is vulnerable to salt stress [36]. Furthermore, potato suffers damage at –3 °C and has no ability to acclimate to cold conditions [36]. Traditional potato breeding is very difficult because of the complicated genetic background and heterozygosity of the plant [37]. Therefore, the use of genetic engineering to improve the tolerance of potato to abiotic stress is essential for future cultivation efforts. It has been reported that overexpression of *StNF-YA* enhanced drought tolerance in potato and that overexpression of the *AtHKT1* gene is able to improve potato salt tolerance [38,39]. In addition, overexpression of *StEREBP1* enhanced tolerance to cold and salt stress in transgenic potato plants [40]. Although the function of CIPKs has been rigorously studied in *Arabidopsis*, a systematic and thorough identification of the *CIPK* gene family is lacking in potato; consequently, the function of CIPKs in potato is largely unknown. In fact, the expression pattern of *StCIPK* genes under different abiotic stresses is unknown, and it is in doubt whether the overexpression of a single *StCIPK* gene increases potato drought and osmotic tolerance. Potato is vulnerable to environmental stresses; therefore, analyzing the response mechanism and function of potato CIPK in extreme environments is of great importance.

In this study, 27 *StCIPK* genes were identified using the Hidden Markov Model (HMM) and BLAST search. In addition, genomic information, chromosomal location, gene duplication, cis-acting regulatory elements and the structure of *StCIPK* genes were analyzed. Phylogenetic relationships and conserved domains of StCIPKs were also investigated. The expression profiles of *StCIPK* genes were determined in different tissues and under different abiotic stress treatments. Furthermore, the regulatory function of StCIPK10 was examined in drought and osmotic stress tolerance and ABA sensitivity, as was the interaction between CBLs and CIPK10.

The results of this study enhance our understanding of the role of the CBL–CIPK module in potato and provide a reference for further research into improving the quality and resistance of potato.

## 2. Results

### 2.1. Identification, Gene Duplication and the Phylogenetic Tree of StCIPK Family Genes

Using the NAF domain (PF03822) as a reference sequence, the potential members of the *StCIPK* gene family were identified using an HMM search. A total of 26 AtCIPKs were used as query sequences to search the potato protein database using BLASTP. Results from both search methods were compiled and redundant sequences were removed from the data set, leading to the identification of 27 *StCIPK* sequences. Results of InterPro and SMART analyses indicated that each StCIPK amino acid sequence contained the NAF and protein–protein interaction (PPI) domains. The 27 *StCIPK* genes were named *StCIPK1* through to *StCIPK27*, according to their locations on various chromosomes. The chromosomal distribution of these genes was uneven (Figure S1), with one *StCIPK* gene on chromosomes 1, 4, 8, 10 and 11, two on chromosomes 3, 7 and 9, three on chromosomes 2 and 5, four on chromosome 6, and six on chromosome 12 (Table 1). Proteins encoded by the *StCIPK* genes varied in length from 370 to 531 amino acids (aa) (Table 1). The predicted molecular mass (MW) of StCIPK proteins varied from 42 to 60 kDa, and their isoelectric point (pI) ranged from 5.97 to 9.36 (Table 1). This diversity in protein properties implies that StCIPKs exhibit a wide range of biological effects.

**Figure 1:**
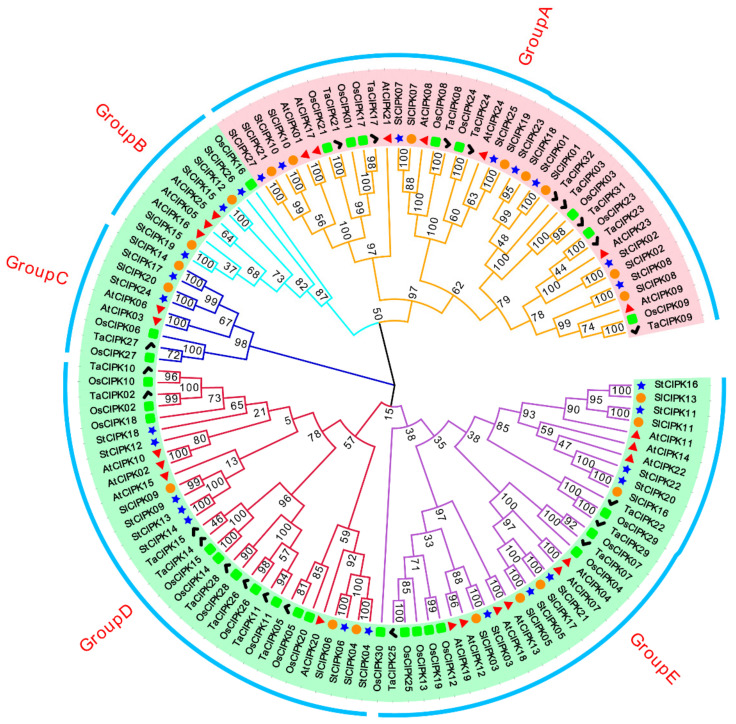
Phylogenetic relationships among plant CIPKs. Amino acid sequences of CIPKs were used to reconstruct a maximum likelihood phylogenetic tree using MEGA7 with the maximum likelihood method and 1000 bootstrap test replicates. At, *Arabidopsis thaliana* (red triangle); Os, *Oryza sativa* (green square); Ta, *Triticum aestivum* (black check mark); Sl, *Solanum lycopersicum* (orange circle); St, *Solanum tuberosum* (blue star). Red background indicates intron-rich *CIPK* genes; green background indicates intron-poor genes.

To study the evolutionary relationships among *CIPK* genes of potato and other plant species, a maximum likelihood phylogenetic tree was reconstructed using CIPK amino acid sequences. This revealed that all CIPKs could be divided into five groups: A, B, C, D and E (Figure 1). StCIPK01/02/07/08/10/23/25/27 were clustered in group A; StCIPK15/19 /26 in group B; StCIPK17 and StCIPK24 in group C; StCIPK04/06/09/12/13/14/18 in group D; and StCIPK03/05/11/16/20/21/22 in group E. Most of the StCIPKs were closely related to SlCIPKs, with the exceptions of StCIPK12 and StCIPK18, which showed the closest phylogenetic relationship with AtCIPK10 and OsCIPK18, respectively. These results not only reflect close evolutionary relationships between *StCIPK* and *SlCIPK* genes but also the existence of a common ancestor of potato and tomato *CIPK* genes. In addition, the intron-rich *StCIPKs* were clustered in group A, while the intron-poor and intron-free *StCIPKs* were clustered in the other four groups. These results are consistent with those obtained in *Arabidopsis*, maize (*Zea mays* L.), turnip (*Brassica rapa* subsp. *rapa*) and grapevine.

To further explore the evolutionary history of *StCIPK* genes, *CIPK* gene duplications in potato and *Arabidopsis* were investigated. Six duplication events were detected among 12 *StCIPK* genes, including one tandem duplication (*StCIPK13*/*14*) and five segmental duplications (*StCIPK01*/*23*, *StCIPK04*/*06*, *StCIPK10*/*27*, *StCIPK11*/*16* and *StCIPK17*/*24*) (Figure S1). Furthermore, to explore potential functional differences among *StCIPKs*, non-synonymous (Ka) and synonymous (Ks) substitution rates were determined, as was the Ka/Ks ratio using KaKs_Calculator2.0. The divergence time (T) of all six pairs of *StCIPK* paralogs was estimated using the formula T = Ks/2r, where r = 1.5 × 10^−8^ synonymous substitutions per site per year for dicotyledonous plants [41]. The Ka/Ks ratio of the *StCIPK13*/*14* tandem duplication was 0.73, while those of the five *StCIPK* segmental duplications ranged from 0.06 to 0.33 (mean value = 0.18) (Table S1). The tandem duplication event of *StCIPK* genes occurred approximately 17 million years ago (MYA), and the segmental duplication events occurred 28–52 MYA (mean value = 46 MYA). Thus, the six pairs of *StCIPK* paralogs were under purifying selection. However, the Ka/Ks ratio of one *StCIPK* paralogous gene pair (*StCIPK01/23*) was relatively large (0.33), suggesting that *StCIPK01* and *StCIPK23* evolved from a common ancestor. Since the function of many *AtCIPK* genes has been identified, analysis of potato homologs of *AtCIPK* genes may help elucidate their function. Ten *CIPK* orthologous gene pairs were identified between potato and *Arabidopsis*, and the Ka/Ks ratio of these genes ranged from 0.05 to 1.23 (mean value = 0.32) (Figure S1 and Table S1). This implies that the conserved linkage arrangements between potato and *Arabidopsis* diverged from a common ancestor 45–205 MYA. The results show that the majority of potato and Arabidopsis *CIPK* orthologs were under strong purifying selection; however, one *CIPK* orthologous gene pair (*StCIPK16*/*AtCIPK14*) was under positive selection (Ka/Ks > 1).

### 2.2. Analysis of Gene Structure and Cis-Acting Elements 

Gene structure analysis showed that 19 *StCIPKs* contained no more than three introns and the other eight *StCIPKs* contained no fewer than ten introns (Figure S2). These results, together with the results of phylogenetic analysis (Figure 1), indicate that *StCIPKs* with a similar number of introns are highly conserved. The conserved domain of StCIPK proteins were analyzed using MEME software (version 5.0.3). Ten motifs were identified (Figure S2 and Table S2). All StCIPKs, except StCIPK13 and StCIPK14, contained the NAF–FISL motif, which plays an important role in regulating the interaction between CIPKs and CBLs [9]. Amino acid sequence alignment showed that the NAF–FISL motif was highly conserved at amino acid positions 5 (asparagine; N), 6 (alanine; A) and 7 (phenylalanine; F) and relatively conserved at positions 10 (isoleucine; I), 13 (serine; S), and 18 (leucine; L). On account of the fact that the fifth amino acid residue in the NAF–FISL motif of StCIPK13 and StCIPK14 was an S instead of an N (Figure S3), it is not shown separately in Figure S2. Similar variation has also been detected in the CIPKs of other plant species, such as AtCIPK4, BnaCIPK7 and BrrCIPK4 [28,33,37,42]. The PPI motif was identified at the *C*-terminal region of all 27 StCIPKs (Figure S2). This has been shown to mediate the phosphorylation of CIPK [43]. However, the amino acid sequence of the PPI motif showed limited conservation at positions 5 (arginine; R), 6 (F), 7 (threonine; T) and 8 (S) (Figure S3).

To further understand the potential regulatory mechanism of *StCIPK* genes, the cis-acting elements in a 1.5 kb sequence upstream of the transcription start site of each gene were searched using PlantCARE software (Model Version: 1). A large number of cis-acting elements were identified. Except for the basic gene expression control elements (CAAT and TATA), 50 cis-acting elements were divided into four groups according to their biological function (Figure S4 and Table S3). Group 1 contained light-responsive elements, such as G-box, SP1, GATA-motif, AE-box and others. Group 2 contained hormone-responsive elements, including O2-site, AuxRR-core, GTGGC-motif, CGTCA-motif, TGACG-motif, TGA-element, TGA-box, TCA-element, *p*-box, TATC-box, AT-rich sequence and ABA-responsive element (ABRE). ABREs were found in the promoter regions of most *StCIPK* genes, except *StCIPK05*/*06*/*13*/*18*/*19*/*23*. Group 3 contained plant growth and development-responsive elements, including RY-element, CAT-box, GCN4-motif, HD-Zip1 and MSA-like. Group 4 contained abiotic stress-responsive elements, such as MYB and low temperature-responsive (LTR) element. The MYB element, which is involved in plant defense against drought stress, was found in all *StCIPK* genes except *StCIPK04*/*11*/*14*/*26*. The LTR element was detected in the promoters of *StCIPK02*/*03*/*12*/*13*/*18*/*23*/*25*. Other abiotic stress-responsive elements, such as MBS, WUN-motif, ARE, GC-motif, TC-rich repeats, MYC and dehydration-responsive element (DRE), were also detected in *StCIPK* gene promoters.

### 2.3. Expression Analysis of StCIPK Genes in Different Tissues

**Figure 2:**
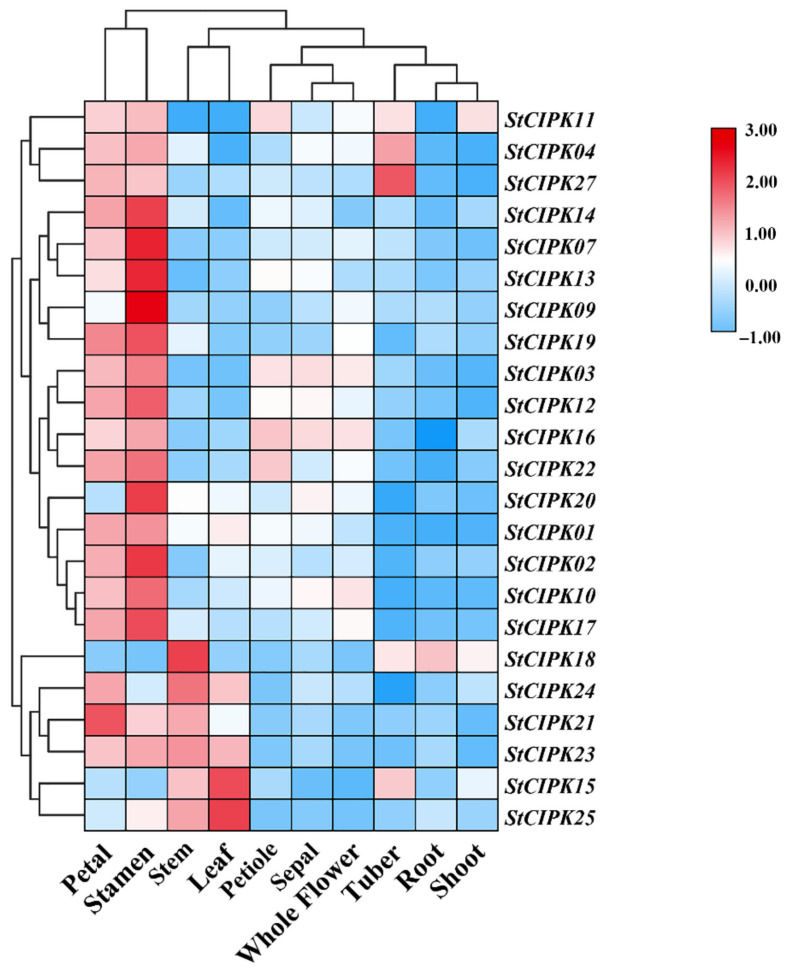
Heatmap showing the expression profiles of *StCIPK* genes in different tissues. Gene expression was analyzed by qRT-PCR. Three biological replicates, each containing three technical replicates, were conducted for each gene. Relative transcript levels were calculated using the comparative threshold (2^−ΔΔCT^) method and normalized using log2.

The expression of *StCIPK* genes was analyzed in 10 different tissues of potato plants—root, leaf, stem, shoot, tuber, sepal, stamen, petiole, petal and whole flower—using quantitative reverse-transcription PCR (qRT-PCR). Gene expression data in different tissues is shown as a heatmap in Figure 2. Four *StCIPK* genes, *StCIPK05*/*06*/*08*/*26*, showed no expression (Ct value > 35). The remaining 23 *StCIPK* genes were expressed in at least one tissue. For example, *StCIPK15*/*18*/*21*/*23*/*24*/*25* were highly expressed in stems; *StCIPK15*/*23*/*24*/*25* were highly expressed in leaves; *StCIPK04*/*11*/*15*/*18*/*27* showed high expressions in tubers. In petals, all genes showed high expression, except *StCIPK09*/*15*/*18*/*20*/*25*. The *StCIPK18* gene showed the highest expression in roots. High transcript levels were observed for *StCIPK03*/*11*/*16*/*22* in the petiole. All *StCIPK* genes showed high expression in stamens, except *StCIPK15*/*18*/*24*. The *StCIPK03* and *StCIPK16* genes displayed similar transcript levels in all tissues. In addition, high transcript levels of *StCIPK11* and *StCIPK18* were detected in shoots.

### 2.4. Expression Analysis of StCIPK Genes under Different Abiotic Stresses and ABA treatment

**Figure 3:**
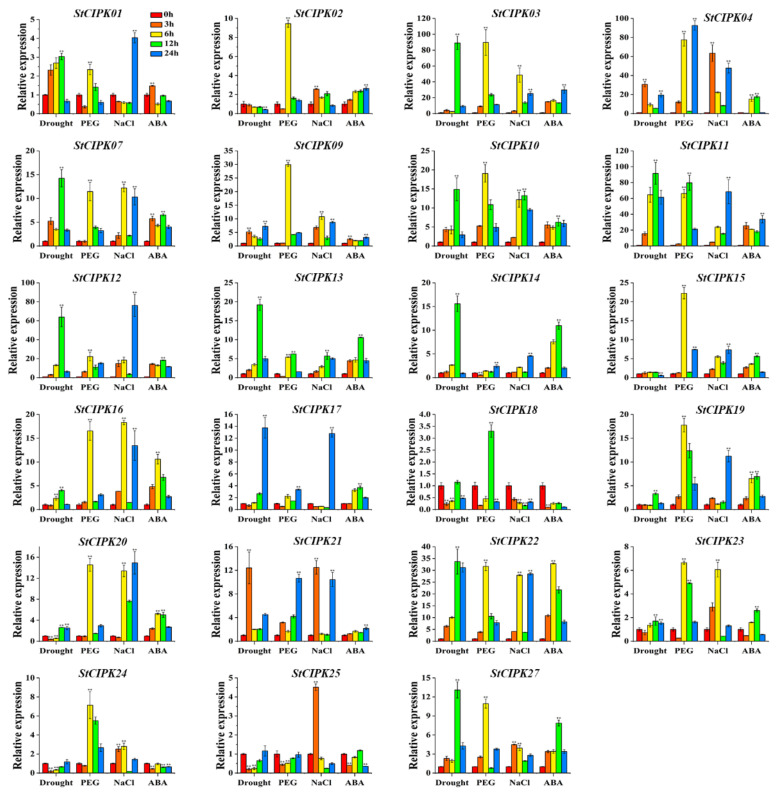
Expression profiles of *StCIPK* genes in response to drought, PEG and salt stresses and ABA treatment. Data are mean ± SD (*n* = 3). Significance compared with 0 h was determined using the ANOVA Duncan’s test; *p* < 0.01, highly significant (**).

To obtain further information about the potential function of *StCIPK* genes, their expression patterns were investigated under 24 h of drought, polyethylene glycol (PEG) or salt stress or ABA treatment (Figure 3). *StCIPK05*/*06*/*08*/*26* showed no expression (CT values > 35) under the different treatments tested. Under drought stress, *StCIPK03*/*04*/*07*,/*09*/*10*/*11*/*12*/*13*/*21*/*22*/*27* were up-regulated throughout the experiment, whereas *StCIPK16*/*17*/*19*/*23* were up-regulated only at one or two time points compared with 0 h. With prolonged treatment time, the expression of *StCIPK02* was gradually down-regulated under drought stress. *StCIPK20*/*24*/*25* showed similar expression patterns under drought stress: they were down-regulated compared with 0 h at the 3 h and 6 h time points but up-regulated at the 12 h and 24 h time points. Under PEG stress, the expression levels of seven *StCIPK* genes (*StCIPK03*/*10*/*12*/*16*/*19*/*21*/*22*) were up-regulated compared with 0 h at all time points during treatment, whereas those of *StCIPK25* were down-regulated. In addition, the expression patterns of *StCIPK02/13*/*23* were relatively similar under PEG stress; all of these genes were down-regulated compared with 0 h at the 3 h time point but up-regulated at the other three time points. Under salt stress, transcript levels of *StCIPK04*/*09*/*11*/*14*/*15*/*16*/*22*/*27* were up-regulated compared with 0 h at all treatment time points, whereas those of *StCIPK18* were down-regulated. Moreover, expression levels of *StCIPK01* and *StCIPK17* were down-regulated at 3, 6 and 12 h compared with 0 h but up-regulated at 24 h under salt stress. Expression of *StCIPK18*/*24*/*25* was suppressed by ABA; compared with the control (0 h), the expression level of *StCIPK18* decreased by approximately 80% in the presence of ABA. Overall, transcript levels of most *StCIPK* genes were up-regulated under ABA treatment at two or more time points.

### 2.5. StCIPK10 Positively Modulates Responses to Drought and Osmotic Stress in Potato

**Figure 4:**
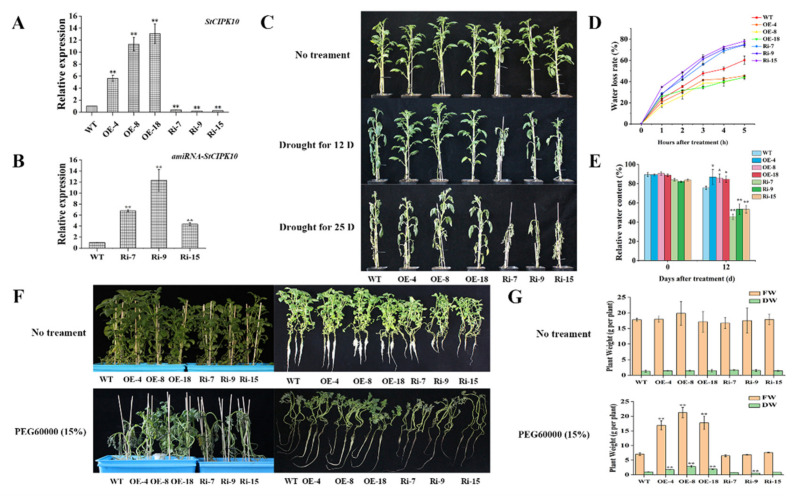
*StCIPK10* positively modulates responses to drought and osmotic stress in potato. (**A**) Transcript levels of *StCIPK10* in transgenic and WT potato plants. (**B**) Transcript levels of amiRNA-*StCIPK10* in RNAi and WT plants. (**C**) Drought tolerance phenotypes of transgenic and WT potato plants in pots filled with soil: vermiculite mixture (3:1) after 12 d and 25 d of withholding water. (**D**) Water loss rates of transgenic and WT potato plants over 5 h. (**E**) RWC of transgenic and WT potato plants after 12 d of withholding water. (**F**) Phenotypes of transgenic and WT potato plants incubated in half-Hoagland solution without stress or with 15% PEG6000. (**G**) FW and DW of transgenic and WT potato plants without stress or after 15% PEG6000 treatments. FW, fresh weight; DW, dry weight. Experiments were repeated three times independently. Data are mean ± SD (*n* = *3*). Significance compared with WT was determined using the ANOVA Duncan’s test; *p* < 0.05, significant (*); *p* < 0.01, highly significant (**).

CIPKs participate in most calcium signaling pathways and also play a critical role in plant responses to abiotic stresses, including drought and osmotic stresses [43]. Our data confirmed that drought and PEG stress induced and enhanced the transcription levels of *StCIPK10*. The *AtCIPK01* sequence is homologous with *StCIPK10*, and loss of *AtCIPK1* function has already been proven to impair osmotic stress responses in plants (Figure 1 and Figure S1) [19]. Consequently, *StCIPK10*-overexpressing or amiRNA-silenced potato plants were produced to investigate the potential function of *StCIPK10* in drought and osmotic tolerance. Amplification of *HygR* (566 bp) and *NPTII* (676 bp) using RT-PCR confirmed that the recombinant plasmids were integrated into the DNA of plants (Figure S5C,D). Nineteen *StCIPK10*-overexpression lines and 17 RNAi lines of potato were obtained (Figure S5A–C), and three *StCIPK10*-OE lines (OE-4, OE-8 and OE-18) and three *StCIPK10*-RNAi lines (Ri-7, Ri-9 and Ri-15) were selected for further analysis by qRT-PCR. Transcription levels of *StCIPK10* in OE-4, OE-8 and OE-18 lines were increased significantly over those in the wild type (WT) (Figure 4A). Transcription levels of *StCIPK10* in Ri-7, Ri-9 and Ri-15 decreased significantly, while those of amiRNA-*StCIPK10* were increased significantly compared with those in the WT (Figure 4A,B). Additionally, one important target was validated using modified 5′ RLM-RACE with amiRNA-*StCIPK10* cleavage sites (Figure S5E).

To investigate the effect of *StCIPK10* overexpression or RNAi expression on drought tolerance, 6-week-old transgenic and WT potato plants were deprived of water for 25 d. After 12 d of withholding water, the OE lines showed visibly better drought tolerance than WT or RNAi lines. Both WT and transgenic plants shriveled after 25 d of withholding water. However, wilting of WT and RNAi lines was more serious, and RNAi plants even began to dry up (Figure 4C). Water loss rates and the relative water content (RWC) of leaves can also reflect the drought tolerance of plants. OE lines had the lowest water loss rates, with RNAi lines having the highest (Figure 4D). After 12 d of drought treatment, the RWC of OE lines was 10% higher, on average, than that of WT lines and 30% higher than that of RNAi lines (Figure 4E).

Four-week-old transgenic and WT potato plants grown on MS medium were incubated in transplanting boxes with half-Hoagland solution in a growth chamber. After 3 weeks, the nutrient solution in each box was supplemented with 15% PEG6000 or with no treatment for 1 week. All plants receiving no osmotic treatment grew normally (Figure 4F). Under PEG stress, OE-4, OE-8 and OE-18 lines maintained good growth; in contrast, Ri-7, Ri-9, Ri-15 and WT lines dried up and died (Figure 4F). There was no significant difference in FW or DW between WT and transgenic plants under no treatment; however, the FW and DW of OE lines were larger than those of WT and RNAi lines under PEG treatment (Figure 4G).

**Figure 5:**
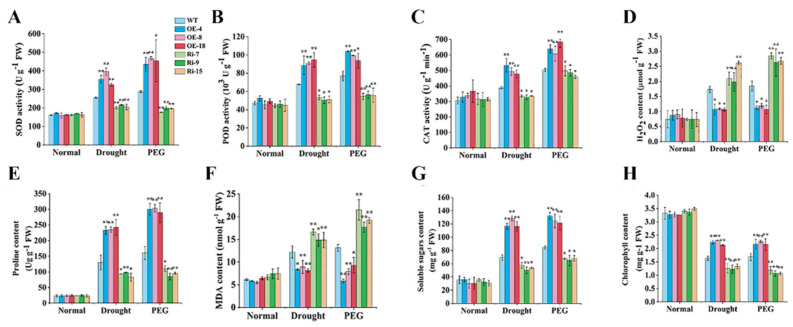
Analysis of physiological indices of transgenic and WT potato plants under drought and PEG6000 treatment. (**A**) SOD activity, (**B**) POD activity, (**C**) CAT activity (**D**) H_2_O_2_ content, (**E**) proline content, (**F**) MDA content, (**G**) soluble sugars content and (**H**) chlorophyll content in leaves. Experiments were repeated three times independently. Data are mean ± SD (*n* = *3*). Significance compared with the no-stress treatment was determined using the ANOVA Duncan’s test; *p* < 0.05, significant (*); *p* < 0.01, highly significant (**).

To explain the above differences, several physiological indices (SOD, POD and CAT activity, H_2_O_2_, proline, MDA, soluble sugars and chlorophyll content) were tested in the transgenic and WT potato plants after drought and PEG treatments, respectively. SOD activity, POD activity, CAT activity, proline content, soluble sugar content and chlorophyll content were higher in *StCIPK10*-OE lines than in the WT, while H_2_O_2_ and MDA content were lower in *StCIPK10*-OE lines compared with WT lines (Figure 5). By contrast, *StCIPK10*-RNAi lines showed opposite trends (Figure 5).

### 2.6. Overexpression of StCIPK10 Increases ABA Sensitivity and Stomatal Closure in Transgenic Potato

**Figure 6:**
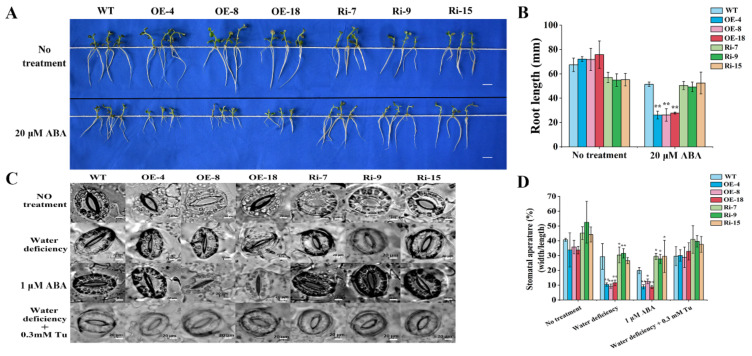
Root lengths and stomatal movements of *StCIPK10* transgenic and WT lines. (**A**) Seedlings grown on 1/2 MS medium and 1/2 MS medium with 20 µM ABA for 14 days. Bars = 1 cm. (**B**) Root length of *StCIPK10* transgenic and WT seedlings. (**C**) Stomatal closure under water deficiency, ABA and TU treatments. Bars = 20 µm. (**D**) Statistical analysis of stomatal width/length ratio. Experiments were repeated three times independently. Data are mean ± SD (*n* = *3*). Significance compared with no treatment was determined using the ANOVA Duncan’s test; *p* < 0.05, significant (*); *p* < 0.01, highly significant (**).

Up-regulation of *StCIPK10* in response to drought, PEG and exogenous ABA suggested a possible role of StCIPK10 in abiotic stress signal transduction pathways mediated by ABA. When seedlings of equal length were grown on 1/2 MS for 14 days, OE plants produced longer roots than WT or RNAi plants (Figure 6A,B). This indicates that StCIPK10 has a positive effect on the growth of roots. Growth of WT and OE plants was inhibited by ABA, with shorter root length in OE plants than in WT plants (Figure 6A,B). Overexpression of StCIPK10 therefore increases potato sensitivity to ABA.

Under drought stress, OE lines showed better drought tolerance and water status than WT or Ri lines (Figure 6C,D). Transpiration through stomata plays a leading role in water loss from plants under drought conditions. Therefore, to determine whether StCIPK10 affects stomatal closure, the stomatal conductance (width/length ratio) of leaves was determined under different conditions. The stomata of all lines were fully open in buffer solution in the light (Figure 6C,D). When leaves of all lines were exposed to the air for 2 h, OE lines displayed lower stomatal width/length ratios. We also investigated whether StCIPK10 regulation and control of stomatal closure is mediated by ABA. When leaves were floated in buffer solution with 1 µM ABA for 2 h, OE lines had lower stomatal width/length ratios than WT and Ri lines (Figure 6C,D). In addition, when the leaves were pretreated by ABA biosynthesis (tungstate sodium, TU) for 2 h before exposure to air for 2 h, water deficiency-induced stomatal closure was inhibited, and all lines showed comparable mean stomatal apertures. These results indicate that the participation of StCIPK10 in stomatal closure is mediated by ABA.

### 2.7. StCIPK10 Regulates Expression of Stress-Induced Genes in Transgenic Potato

**Figure 7:**
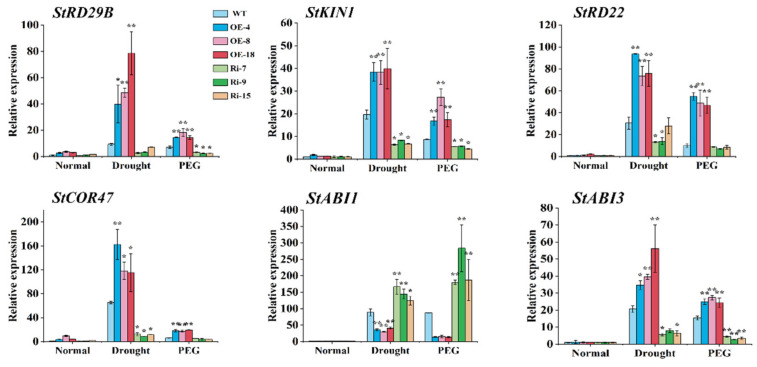
Expression levels of drought- and ABA-responsive genes are altered in WT and transgenic potatoes under drought and PEG stress. Data are mean ± SD (*n* = *3*). Significance compared with normal conditions was determined using the ANOVA Duncan’s test; *p* < 0.05, significant (*); *p* < 0.01, highly significant (**).

Plant responses to abiotic and ABA stress are specific and result in drastic induction of stress gene expression in cells. The expression levels of six drought-, osmotic- and ABA-responsive marker genes were analyzed using qRT-PCR: *StRD29B*, *St*KIN1, *StRD22*, *StCOR47*, *StABI1* and *StABI3* [44,45,46,47]. Under no treatment, none of the genes examined showed differences in expression patterns between the WT and *StCIPK10* transgenic potatoes. Upon drought and PEG treatment, expression of all genes was induced in both WT and *StCIPK10* transgenic plants (Figure 7). Drought- and PEG-mediated induction of all genes except for *StABI1* in the OE lines was significantly stronger than induction in WT and RNAi lines; however, expression of *StABI1* was significantly enhanced in RNAi lines compared with that in OE and WT lines. These results suggest that *StCIPK10* improves drought tolerance in potato by regulating the expression of stress- and ABA-responsive genes.

### 2.8. Interaction between StCIPK10 and StCBL Proteins

**Figure 8:**
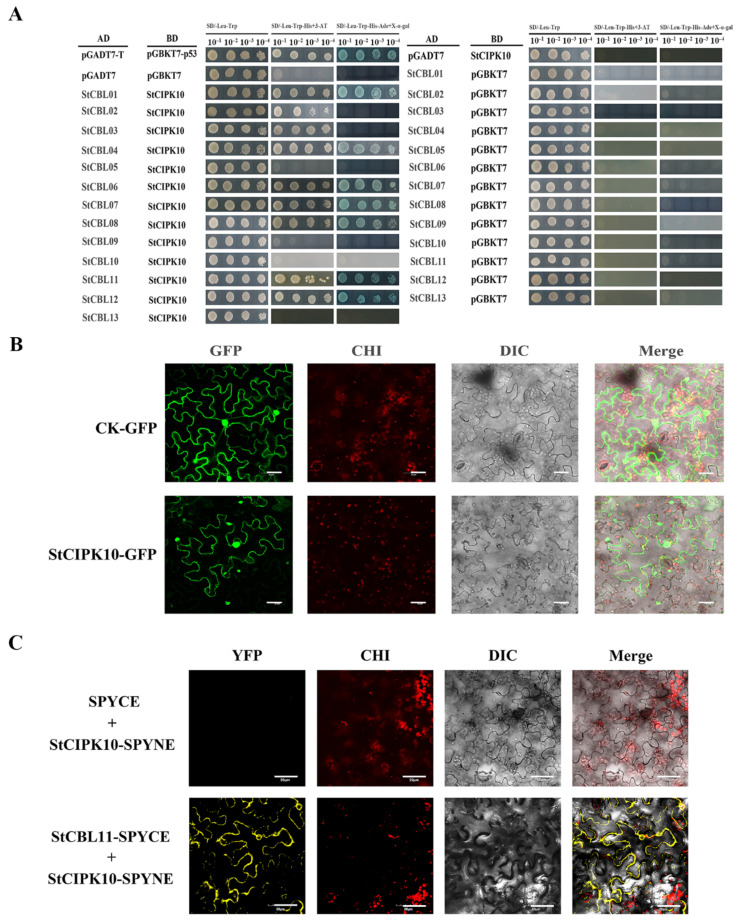
Interaction between StCIPK10 and StCBLs demonstrated by yeast two-hybrid assay and BiFC. (**A**) Y2H assay analyzing the interaction between StCIPK10 (bait) and StCBLs (prey). The pGADT7-T and pGBKT7-p53 pair was used as a positive control. Transformants were spotted separately on SD/-Leu/-Trp medium, SD/-Leu/-Trp/-His medium with 10 mM 3-AT and SD/-Leu/-Trp/-His/-Ade medium with X-α-gal. Each colony was resuspended in 10 μL sterile water and then diluted from 10^−1^ to 10^−4^. At least three colonies per vector combination were tested. (**B**) Subcellular localization analysis of StCIPK10-GFP in *Nicotiana benthamiana* leaves. An empty vector (GFP) served as a control. Bars = 30 µm. (**C**) BiFC assay of the interaction between StCIPK10 and StCBL11. StCIPK10 was introduced into the pSPYNE vector and fused with *N*-terminal YFP; StCBL11 was introduced into the pSPYCE vector and fused with *C*-terminal YFP. YFPc + StCIPK11–YFPn was used as a negative control. CHI: chloroplast; DIC: bright light. Bars = 50 µm.

Promiscuous interaction between CBLs and CIPKs to form a module plays different roles in response to different abiotic stresses in plants. Therefore, StCIPK10 is assumed to interact with one or multiple CBL proteins in potato. Thirteen StCBL proteins were identified and cloned for yeast two-hybrid interaction analysis with StCIPK10 (Table S4) [48]. StCIPK10 interacted with StCBL1, StCBL4, StCBL6, StCBL7, StCBL8, StCBL11 and StCBL12 (Figure 8A). Following analysis of homology between StCBLs and AtCBLs, StCBL11 was chosen for further study. Subcellular localization and bimolecular fluorescence complementation (BiFC) experiments were used for verifying the interaction between StCIPK10 and StCBL11 in vivo. Subcellular localization analysis showed that both GFP-StCIPK10 and control GFP were mainly localized to the nucleus, cytoplasm and membrane (Figure 8B). Strong yellow fluorescent protein (YFP) signal revealed that StCBL11 interacts with StCIPK10 in the cell membrane (Figure 8C). The above results indicated that StCIPK10 is located to the cell membrane in vivo by StCBL11.

## 3. Discussion

Plants have evolved complex regulatory mechanisms to adapt to harsh environmental conditions. CBL–CIPK is an important sensor in the Ca^2+^ signaling pathway which contributes to the maintenance of normal plant growth and the regulation of environmental stress responses. The *CIPK* gene family has been studied in the model plant Arabidopsis and in several different crops, including rice, eggplant and others, yet very few studies have been conducted with potato [28,29,30,31,32,33]. Here, the HMM and BLAST searches were used to identify 27 *StCIPK* genes in the potato genome database based on conserved domains and CIPK sequences of Arabidopsis. A comprehensive analysis of their chromosomal distribution, evolutionary relationships, exon–intron structures, conserved domains, cis-acting elements, spatial expression patterns and abiotic stress sensitivity were conducted. The *StCIPK10* gene was cloned, which is induced by drought, PEG and exogenous ABA, and its biological functions against drought and osmotic stress in potato were characterized, revealing its possible mechanism. This is the first study to our knowledge to document the function and mechanism of *StCIPK10* in potato.

### 3.1. StCIPK Gene Expansion and Evolution in Potato

As shown in Figure S1, *StCIPK* genes are randomly distributed on all 12 potato chromosomes, but most are clustered toward the chromosome ends. The uneven distribution of *StCIPK* genes on chromosomes indicates that they may have been subject to heritable variation during potato evolution. Phylogenetic analysis divided all *StCIPK* genes into five groups. Interestingly, intron-rich *StCIPK* genes were clustered in group A, and intron-poor genes were distributed in the other four groups (Figure 1 and Figure S2). Intron–exon organization and number of introns were typical markers of evolution within most gene families [49]. In eukaryotes, introns are produced by exon shuffling, which increases the types of genes and the functions of proteins [50]. Similar clustering of Arabidopsis and maize *CIPK* genes has been detected [28,51]. Therefore, it is reasonable to presume that the evolution of *StCIPK* gene structure was promoted by a change in the number of introns, and this change may be related to different environmental stresses. This phenomenon also reveals that similar intron changes promoted the structural evolution of the *CIPK* gene family before the divergence of plants into dicot and monocot lineages [52]. 

Gene duplication, another important evolutionary mechanism, provides materials for mutation, leading to genetic evolution. In this study, six pairs of *StCIPK* gene duplicates were identified, of which one pair was produced by tandem duplication (3%) and five pairs were produced by segmental duplications (18%) (Figure S1). This indicates that segmental duplication events were primarily responsible for the expansion of the *StCIPK* gene family. Furthermore, most duplication events involved intron-poor *StCIPK* genes. A similar phenomenon has been observed in the CIPK gene families of Arabidopsis and soybean (*Glycine max* L.) [28,53]. In addition, the Ka/Ks ratio of all *StCIPK* duplicate pairs was less than 1, suggesting that the *StCIPK* gene duplicates were under purifying selection (Table S1). 

As the typical conserved domain of CIPKs, the NAF–FASL motif mediates interactions between CBLs and CIPKs, as well as interaction between CIPKs and type 2C protein phosphatases (PP2C) through the PPI motif [54]. The NAF–FISL motif in all StCIPKs was detected, except StCIPK13 and StCIPK14 (Figure S2). Amino acid sequence alignment showed that the NAF–FASL motifs of StCIPK13 and StCIPK14 contained an S (serine) at the 5 aa position instead of an N (asparagine) (Figure S3). Similar variation has also been reported in *Arabidopsis* and turnip *CIPK* genes [28,55]. Previous research shows that the form of combination between CBLs and CIPKs is the basis of how CBL–CIPK modules respond to various environment stress [56]. Therefore, this amino acid variation in the NAF–FISL motif may result in functional divergence among *StCIPK* genes.

### 3.2. StCIPK10 is a Positive Regulator of Potato Tolerance to Drought and Osmotic Stress of Potato

The expression levels of StCIPK genes in different tissues of potato plants were examined using qRT-PCR. The results revealed differences in StCIPK expression levels in different tissues. Most StCIPK genes were highly up-regulated in the petals and stamen, indicating that StCIPK genes likely play a key role in pollen germination. Functional characterization of AtCBL1 and AtCBL9 confirmed that CIPKs also participate in the process of pollen germination; however, which specific CIPKs are involved in this process is a question requiring further investigation with controlled trials. In addition, StCIPK18 showed higher expression levels in the stem and root than in other tissues, indicating a potential function of StCIPK18 in the genetic transformation of potato. The diverse expression patterns of StCIPK genes in different tissues indicate that each gene plays a different role in the growth and development of potato plants.

Previous research shows that CIPKs play a vital role in the response to multiple abiotic stresses [57]. AtCIPK24 was proven to enhance the salt tolerance of Arabidopsis plants by activating the tonoplast-localized antiporter NHX [16]. In this study, *StCIPK25*, which is homologous to *AtCIPK24*, was up-regulated under salt treatment, and the stress-related cis-acting element MYB was detected in its promoter region (Figure 1, Figure 3 and Figure S4). In Arabidopsis, AtCBL1/9–AtCIPK23 enhance the absorption of K^+^, leading to stomatal closure in leaves, which indirectly improves drought tolerance [18]. The potato homolog of *AtCIPK24*, *StCIPK02*, showed higher expression levels in drought-treated plants than in control plants, and harbored the drought-related cis-acting element MBS in its promoter region (Figure 1, Figure 3 and Figure S4). Transcription levels of *StCIPK10* and *StCIPK27* were highly up-regulated by all treatments, and both these genes contained the ABRE motif in their promoter regions (Figure 3 and Figure S4). Moreover, *AtCIPK1*, the Arabidopsis homolog of *StCIPK10* and *StCIPK27*, has been shown to respond to multiple abiotic stresses through the ABA signaling pathway [19]. Thus, phylogenetic and gene expression analyses helped elucidate the potential functions of *StCIPK* genes. Moreover, these results provide a theoretical basis for the identification of stress-related genes.

The identification of stress-related cis-acting elements, such as ABRE, MBS, MYB and MYC, in the promoter regions of most *StCIPK* genes explains why *StCIPK* genes are sensitive to abiotic stresses. The plant abiotic stress signaling pathway mediated by the CBL–CIPK module is a highly complicated system [58]. *StCIPK* genes arising from segmental duplications showed diverse expression patterns, whereas those derived from tandem duplications showed similar expression patterns (Figure 3). This result is consistent with the expression patterns of *OsCIPK* and *BrrCIPK* genes and indicates that the *CIPK* gene family has undergone extensive expansion through segmental duplications under the influence of abiotic stress. *StCIPK20* expression was induced more strongly under PEG stress than under drought stress, while expression of *StCIPK24* was induced by PEG treatment but repressed by drought treatment (Figure 3). These results suggest that *StCIPK* genes participate in the regulation of PEG and drought stress signals through different molecular mechanisms. Notably, all six *StCIPK* genes belonging to the intron-poor group (*StCIPK03*/*04*/*11*/*12*/*13*/*22*) were significantly up-regulated by all abiotic stress treatments (Figure 3). This suggests that expansion of the intron-poor clade of the *StCIPK* gene family was a defense mechanism against abiotic stress. In summary, the expression patterns of *StCIPK* genes are highly varied under abiotic stress and hormone treatments. We therefore speculate that *StCIPK* genes play a greater role in abiotic stress tolerance than in plant growth and development.

### 3.3. StCIPK10 Is a Positive Regulator of Potato Tolerance to Drought and Osmotic Stress

As shown by qRT-PCR, the expression of *StCIPK10* was significantly induced by drought, PEG6000 and ABA (Figure 3). Transgenic potato overexpressing *StCIPK10* showed stronger tolerance to drought and osmotic stress than WT lines, but RNAi lines showed the opposite phenotype (Figure 4C, F). Favorable water status can represent tolerance of plants under drought, osmotic and high salinity stress [59,60,61]. Leaves of OE lines retained more moisture compared with those of WT lines after drought and PEG treatment; however, the RNAi lines exhibited obvious sensitivity to drought and osmotic stress, with more water loss and lower relative water content of leaves (Figure 4D,E,G). More healthy leaves, a lower rate of water loss and higher relative water content revealed that StCIPK10 enables cells to maintain normal homeostasis and reduce damage to the plasma membrane. Stress induces plant cells to produce a great deal of ROS, which leads to serious oxidative damage, destroys the integrity of membrane systems and reduces enzyme activity [62]. Furthermore, too much MDA can lead to plasmalemma damage, which aggravates the reduction in the stress tolerance of plants [63]. 

Clearing of ROS in plants is dependent on the activity of SOD, POD, CAT and other enzymes [64]. After drought and PEG treatments, the H_2_O_2_ and MDA contents of OE lines were lower than those of WT plants, but the activity of SOD, POD and CAT and the content of chlorophyll were higher than in WT plants; however, RNAi lines showed the opposite trends (Figure 5A–D,F,H). Under water-deficit conditions, plants accumulate beneficial osmoregulation substances, for instance, proline and soluble sugars, which are used to decrease the osmotic potential of cells and protect plasmalemma from the harmful effects of water deficit [65,66]. The proline and soluble sugar levels of OE lines were higher than those in the WT after drought and PEG treatments, but those of the RNAi lines were lower (Figure 5E,G). Therefore, our results demonstrated that overexpression of *StCIPK10* in potato enhances both the ability to scavenge ROS and the content of corresponding osmoregulation substances, thereby strengthening the tolerance of potato to drought and osmotic stress.

### 3.4. StCIPK10 Is a Positive Regulator of ABA-Dependent Responses

ABA is a universal signaling molecule in plants, playing an important role in regulating root development, stomatal closure and stress responses through transcriptional or translational regulation [67,68]. Stomatal closure induced by ABA is the principal mechanism of plant drought tolerance [66]. In this research, ABA induced up-regulation of *StCIPK10* expression. The sensitivity of stomatal closure to drought and ABA treatment was increased by overexpression of *StCIPK10* in potato, accelerating stomatal closure, while RNAi of *StCIPK10* reduced such sensitivity (Figure 6C,D). This explains why the OE lines exhibited lower rates of water loss and higher RWC than the WT under drought treatment. Overexpression of *StCIPK10* also increased the sensitivity of root growth to ABA treatment (Figure 6A,B). The increased sensitivity of stomatal closure disappeared and the rate of stomatal closure was reduced when leaves of OE lines were treated with the ABA biosynthesis inhibitor Tu prior to water-deficiency treatments (Figure 6C,D).

Expression of the ABA-responsive genes *StRD29B*, *St*KIN1, *StRD22* and *StCOR47* was significantly up-regulated in *StCIPK10* OE lines under drought and PEG stress, reflecting the importance of ABA in *StCIPK10* transgenic potato. ABI signaling proteins are potential targets of the CBL–CIPK molecule; ABI1 and ABI2 are negative regulators of ABA responses, while ABI3, ABI4 and ABI5 function as positive regulators of ABA responses. Recent studies have confirmed claims that high expression levels of ABI3, ABI4 and ABI5 can induce an ABA hypersensitive response in *Arabidopsis thaliana* [47]. In our study, *StABI3* expression levels in *StCIPK10*-OE lines were higher than those in the WT and *StABI1* expression levels were lower than those in the WT under drought and PEG treatment, while RNAi lines showed the opposite trends. Thus, StCIPK10 is located at an intersection between the ABA signaling pathway and the abiotic stress signaling pathway, controlling both root growth and stomatal closure in potato.

### 3.5. Interactive Mechanism of StCIPK10 with Upstream Regulators 

The CBL–CIPK module represents a large and complicated system in plant cells, and diversification of the interaction between CBLs and CIPKs guarantees various functions of CIPKs in plants. The specific interaction between CBLs and CIPKs is induced by specific stress signals [58]. The CcCIPK14–CcCBL1 pair positively modulates drought tolerance by enhancing flavonoid biosynthesis [27]. The GhCBL2–GhCIPK6 pair modulates plant sugar homeostasis by interacting with the tonoplast sugar transporter TST2 [69]. Therefore, research into the interaction between CBLs and CIPKs in potato is well justified. Our studies using yeast two-hybrid analysis indicate that StCIPK10 interacts with StCBL1, StCBL4, StCBL6, StCBL7, StCBL8, StCBL11 and StCBL12. This is evidence that StCIPK10 really does function in various signal transduction pathways. 

Since the CIPK protein sequence contains no localization motifs, the subcellular localization of CBL–CIPK modules is determined by myristoylated and acylated modification sites in CBLs [58]. In this study, StCBL11 interacted with StCIPK10, specifically recruiting StCIPK10 to the plasma membrane in *Nicotiana benthamiana* cells. This indicates that interaction of StCIPK10 with a potentially myristoylated site of StCBL11 is necessary and sufficient to target StCIPK10 to the plasma membrane. This result also revealed that plasma membrane-localized proteins may be regulated by the StCBL11–StCIPK10 complex. The classic example is the CBL4 (SOS3)–CIPK24 (SOS2) complex, which phosphorylates the Na^+^ transporter SOS1 in the plasma membrane [70]. Moreover, StCIPK10 is located in the nucleus, cytoplasm and plasma membrane. This shows that there is a possibility of StCIPK10 functioning properly in other subcellular localizations when interacting with StCBLs other than StCBL11. There was 79.34% similarity found between AtCBL9 and StCBL11 at the amino acid level. AtCIPK1 shows similarity as high as 64.91% with StCIPK10 at the amino acid level. Research shows that AtCBL9–AtCIPK1 plays an important regulatory role in osmotic stress responses [19]. Therefore, it is necessary to confirm the function of StCBL11 in the potato’s response to drought and osmotic stress.

## 4. Materials and Methods

### 4.1. Identification of StCIPK Genes

Whole-genome data for *Arabidopsis thaliana* and *Solanum tuberosum* were downloaded from Ensembl Plants (http://plants.ensembl.org/index.html, accessed on 10 October 2018). CIPK protein sequences of *Arabidopsis thaliana*, *Oryza sativa*, *Triticum aestivum* and *Solanum lycopersicum* were obtained from Phytozome on the Potato Genome Sequencing Consortium (PGSC) database (http://solan-aceae.plantbiology.msu.edu/, accessed on 10 October 2018). The profile of the NAF domain was downloaded from Pfam (http://pfam.xfam.org/, accessed on 10 October 2018, latest update on 19 November 2021), and HMMER 3.2.1 software was downloaded from HMMER (http://hmmer.org/, accessed on 10 October 2018, latest update on 26 November 2020) [42]. Two methods were adopted for identifying all *StCIPK* genes. First, published AtCIPK protein sequences were used to search candidate StCIPK protein sequences with BLAST (http://blast.ncbi.nlm.nih.gov, accessed on 11 October 2018, latest update on 1 November 2021). Then, the NAF domain (PF03822) from Pfam, which is considered a signature domain of CIPKs, was used to screen candidate StCIPK protein sequences via an HMM search [42]. All protein sequences identified using these two methods were compiled, and redundant sequences were eliminated. All candidate sequences were verified for the presence of the conserved NAF domain using SMART (http://smart.embl-heidelberg.de/, accessed on 11 October 2018, latest update on 26 October 2020) and InterPro (http://www.ebi.ac.uk/interpro/search/sequence/, accessed on 11 October 2018, latest update on 18 November 2021) [71]. ExPASy (https://web.expasy.or-g/prot-param/, accessed on 11 October 2018, latest update on 1 October 2020) was used to analyze the MW and pI of each candidate StCIPK [72].

### 4.2. Chromosomal Localization, Phylogenetic and Gene Duplication Analysis

The PGSC database was used to identify all StCIPKs and to determine the chromosomal locations of the corresponding genes. Chromosomal distribution of *StCIPK* genes was displayed using MapChart software [73]. Multiple sequence alignment of CIPKs was performed using ClustalX1.83 [74]. A phylogenetic tree was constructed using a maximum likelihood estimation in MEGA7, with 1000 bootstrap repetitions to ensure the reliability of internal branches [74]. Gene duplication information for *StCIPK* genes was downloaded from the Plant Genome Duplication Database (PGDD; http://chibba.agtec.uga.edu/duplication/index/locus, accessed on 10 October 2018), and Ka/Ks ratios were calculated using KaKs_Calculator2.0 software [41]. After determining the syntenic relationships between AtCIPKs and StCIPKs, a syntenic map was drawn using CIRCOS software [52].

### 4.3. Cis-Acting Element and Gene Structure Analysis

The promoter regions of *StCIPK* genes were acquired from the Phytozome database. Cis-acting regulatory elements were searched for in the 1.5 kb sequence upstream of the transcription start site using PlantCARE (http://bioinformatics.psb.ugent.be/webtools/p-lantcare/html/, accessed on 13 October 2018), and detected motifs were illustrated using the Gene Structure Display Server (GSDS; http://gsds.cbi.pku.edu.cn/, accessed on 13 October 2018, latest update on 20 April 2021) [75]. *CIPK* genomic sequences were aligned to their corresponding coding sequences in potato. Exon–intron structures of *StCIPK* genes were then obtained and illustrated using GSDS. The MEME website (http://meme-suite.org/, accessed on 15 October 2019, latest update on 25 August 2021) was used to predict conserved domains in StCIPK protein sequences using the following parameters: motif number = 10; motif width = 6–50 aa [76].

### 4.4. Plant Materials and Treatments

Pre-basic seed tubers of the potato cultivar ‘Atlantic’ were sown in pots filled with a soil–vermiculite mixture (3:1), one seed in each pot. The pots were placed in a greenhouse maintained at 21 ± 2 °C with a 16 h light/8 h dark photoperiod and watered once per week. Upon reaching a height of 20 cm, seedlings were subjected to four different treatments. For exogenous ABA treatment, a solution containing 0.1 mM ABA was applied by foliar spray. For salt and osmotic stress treatments, seedlings were irrigated with 200 mM NaCl or 20% PEG6000 (*w/v*), respectively. To induce drought stress, the water content of the soil–vermiculite mixture was limited to 25 ± 5% of soil field capacity. Leaves of all seedlings were collected at 0, 3, 6, 12 and 24 h after each treatment, immediately frozen in liquid nitrogen and stored at −80 °C for subsequent tests. Each sample was collected from three independent seedlings, and three biological replicates were performed.

### 4.5. RNA Isolation and Gene Expression Analysis

Total RNA was extracted from each sample using an RNAsimple Total RNA Kit (Tiangen, China), according to the manufacturer’s instructions. Subsequently, 1 µL of total RNA was used to synthesize first-strand cDNA using a Prime Script RT Reagent Kit (Takara, China), according to the manufacturer’s instructions. qRT-PCR was then performed using a SYBR Green PCR Kit (TaKaRa, Dalian City, Liaoning Province of China, China) in a 20 µL reaction containing 2 μL (100 ng) of cDNA, 10 µL of 2× SuperReal PreMix Plus, 0.4 µL of ROX, 0.6 µL of each primer and 7.8 μL of RNase-free double-distilled water. PCR was performed under the following conditions: initial denaturation at 94 °C for 10 min, followed by 40 cycles of denaturation at 94 °C for 10 s, annealing at 60 °C for 30 s and extension at 72 °C for 40 s. *StEF1a* (PGSC0003DMG400023270) was used as a housekeeping gene for data normalization. All primers were designed using NCBI Primer BLAST and are listed in Table S5. Relative expression levels of *StCIPK* genes were calculated using the 2^−△△Ct^ method [77].

### 4.6. Subcelluar Localization Analysis of StCIPK10

The coding sequence of *StCIPK10* without a stop codon was fused into the vector pCAMBIA1300-35S-EGFP containing enhanced green fluorescent protein (EGFP) driven by the CaMV 35S promoter. The recombinant plasmid was then transferred into *Nicotiana benthamiana* leaf epidermal cells via *Agrobacterium* strain GV3101. The green fluorescence signal was observed at 48 h after injection using a confocal microscope (LSCM800, Zeiss, Oberkochen, Baden-Württemberg, Germany). All were designed using NCBI Primer BLAST and are listed in Table S5.

### 4.7. Protein Interaction Assay

Analysis of interactions between StCIPK10 and StCBLs was performed using the MatchMaker yeast two-hybrid system (Clontech, Dalian City, Liaoning Province of China, China). Coding sequences of 13 StCBL genes were fused, respectively, into the vector pGADT7, and StCIPK10 was fused into the vector pGBKT7. AD-StCBLs and BD-StCIPK10, as well as positive and negative control plasmids, were co-transformed into the yeast strain AH109 using the PEG–LiAc method. Cotransformants were first incubated on double-dropout medium (SD/-Trp/-Leu, SD-LW) for 3 d at 30 °C. For interaction analyses, positive transformants were selected and spotted separately on double-dropout medium (SD-LW), triple-dropout medium (SD/-Trp/-Leu/-His, SD-LWH) with 10 mM 3-AT (3-amino-1,2,4-triazole), or quadruple-dropout medium (SD/-Trp/-Leu/-His/-Ade, SD-LWHA) with X-α-gal by gradient dilution. Plates were incubated at 30 °C. Four days after inoculation, results were recorded and photographed. For bimolecular fluorescence complementation (BiFC) assay, the coding sequence of StCBL11 was fused into the pSPYNE-35S vector and StCIPK10 was fused into the pSPYCE-35S vector, resulting in StCBL11-nYFP and StCIPK10-cYFP. The two vectors were then transferred into *Nicotiana benthamiana* leaf epidermal cells via *Agrobacterium* strain GV3101. The green fluorescence signal was observed at 48 h after injection using a confocal microscope (LSCM800; Zeiss, Oberkochen, Baden-Württemberg, Germany). All primers were designed using NCBI Primer BLAST and are listed in Table S5.

### 4.8. Production of Transgenic Plants

The coding region of *StCIPK10* was fused into the pCAMBIA1300-35S expression vector. In order to silence *StCIPK10*, amiRNA sequences were designed using WMD3 (http://wmd3.weigelworld.or-g/cgi-bin/webapp.cgi, accessed on 15 October 2019, latest update on 1 November 2021). The coding region of *StCIPK10* was first inputted into the WMD3 target search tool to generate a group of amiRNA sequences likely to silence the target gene. One suitable target gene was selected by analyzing the hybridization energy and target gene binding sites of the amiRNA sequences. The WMD3 primer designer tool provided four different oligos. These were used as primers with pRS300, containing the miR319 precursor backbone sequence of miR319a, as a template to amplify the *StCIPK10* amiRNA sequence by overlapping PCR [78]. The StCIPK10-amiRNA sequence was fused into the pBI121-35S expression vector, and each vector was then transferred into potato via *Agrobacterium* strain GV3101 [79]. Independent transgenic potato lines were obtained and identified through hygromycin selection and qRT-PCR analysis. The *StCIPK10* amiRNA sequences and all primers used for vector construction are listed in Table S5. Among the overexpression and RNAi-expression transgenic potato lines, three independent transgenic lines were selected and grown on MS solid medium for several follow-up experiments.

### 4.9. Assays for Drought and Osmotic Stress Tolerance

For water loss assays, leaves of 4-week-old plants from pots were placed in a dish and exposed to air. Loss of fresh weight was determined at 1 h intervals during 5 h in a growth chamber [80]. For drought stress, 6-week-old plants from pots were evaluated for drought tolerance in the growth chamber. Drought phenotypes were determined 12 and 25 days after cessation of watering. For relative water content (RWC) assays, leaf samples (1–2 g) were collected from the third leaf from the top of untreated and drought-treated plants. RWC was calculated as RWC (%) = ((FW − DW)/(SW −DW))*100, where FW, DW and SW represent fresh, dry and saturated weights of leaves, respectively [80]. For osmotic stress, 4-week-old transgenic and WT potato plants grown on MS medium were incubated in transplanting boxes with half-Hoagland solution in the growth chamb ; after 3 weeks, the nutrient solution in the boxes was supplemented with 15% PEG6000 (*w/v*) or no treatment for 1 week. After osmotic stress, growth was observed and photographed, and fresh weight (FW) and dry weight (DW) were recorded. Superoxide dismutase (SOD), peroxidase (POD) and catalase (CAT) activities and proline, malondialdehyde (MDA), soluble sugar and chlorophyll contents were measured using assay kits after drought and osmotic stress (Sinobestbio Biotechnology Co. Ltd., Shanghai, China). Three plants from each line were used, and each experiment was repeated at least three times.

### 4.10. Assays for Stomatal Movement and Phenotype under ABA Treatment

Four-week-old leaves of transgenic and WT potato lines grown in pots were evaluated for stomatal aperture movement in the growth chamber. Stomatal conductance was measured as described previously [78]. Firstly, leaves were immersed in a buffer solution containing 30 mM KCl and 10 mM MES-KOH under light for 4 h until stomata were completely open. To simulate stomatal movement under drought stress, leaves were then exposed to air under light for 2 h. For stomatal movement under ABA treatment, leaves were immersed in a solution containing 1 µM ABA for 2 h. For Tu treatment, the leaves were immersed in the buffer solution with the 0.3 mM Tu, after 2 h incubation, then were exposed to air for 2 h [81]. Stomatal movement was measured and photographed using a microscope trained on epidermal peels of leaves (Primovert, Zeiss, Oberkochen, Baden-Württemberg, Germany). Three plants from each line and three leaves from each plant were used and the length/width ratio of 30 stomata guard cells from each leaf was recorded. MS-grown transgenic and WT potato plant lines were used to determine plant phenotypes under ABA treatment. Apical buds were incubated on MS medium containing 20 µM ABA. After 10 days, the length of plant roots was recorded and photographed. Three plants from each line were used, and all roots from each plant were recorded. 

### 4.11. Validation of amiRNA Target Genes using RLM 5′-RACE

To verify whether the *StCIPK10* gene was degraded by amiRNA, modified RNA Ligase-Mediated 5′ RACE (RLM 5′ RACE) was performed using a FirstChoice^®^ RLM-RACE Kit (Invitrogen). Firstly, the 5′ adaptor was ligated to total RNA and reverse transcribed into cDNA. The outer 5′ RLM-RACE PCR was performed with 5′ RACE gene-specific outer primer and 5′ RACE outer primer using cDNA as the template. Subsequently, the inner 5′ RLM-RACE PCR was performed with 5′ RACE gene-specific inner primer and 5′ RACE inner primer using the product of 5′ RLM-RACE PCR as template. The amplification product was cloned into the pMD18-T vector and sequenced.

### 4.12. Statistical Analysis

Experiments were repeated three times independently. Data are presented as mean ± SD (*n* = 3). Results were analyzed using data variance analysis performed with the ANOVA Duncan’s test. Significance was defined as significant (*) at *p* < 0.05 and highly significant (**) at *p* < 0.01.

## 5. Conclusions

In this study, 27 *StCIPK* genes were identified using HMM and BLAST searches. All *StCIPK* genes were clustered into the five subgroups by phylogenetic inference. qRT-PCR analysis revealed tissue-specific expression patterns for most *StCIPK* genes. Different expression patterns of *StCIPK* genes in response to drought, PEG, NaCl and ABA treatments indicate that these genes potentially function at the intersection of different signaling pathways. Overexpression of *StCIPK10* significantly increased the activation of potatoes’ antioxidant systems and proline contents, while decreasing malondialdehyde enhanced drought and osmotic tolerance. Moreover, overexpression of *StCIPK10* increases ABA sensitivity and results in stomatal closure. StCIPK10 interacts with StCBL1, StCBL4, StCBL6, StCBL7, StCBL8, StCBL11 and StCBL12, and is specifically recruited to the plasma membrane of cells by StCBL11. Taken together, these results provide not only new ideas for increasing potato drought resistance by molecular biological methods, but also for developing potentially superior genetic resources for improving potato varieties.

## Figures and Tables

**Table 1:** Characteristics of *StCIPK* genes and the encoded proteins.

Gene Name	Gene ID	Chr. ^1^	Genomic Location (bp)	CDS Length (bp) ^2^	No. of Exons	Protein Length (aa) ^3^	MW (kDa) ^4^	pI ^5^
*StCIPK01*	Soltu.DM.01G005660.2	1	5,154,274–5,159,709	1317	14	438	50.23	6.72
*StCIPK02*	Soltu.DM.02G002430.1	2	11,576,701–11,584,119	1368	14	455	51.10	8.92
*StCIPK03*	Soltu.DM.02G015750.1	2	31,880,731–31,885,458	1437	1	478	53.76	8.60
*StCIPK04*	Soltu.DM.02G015770.1	2	31,899,161–31,901,152	1377	2	458	52.14	9.05
*StCIPK05*	Soltu.DM.03G000420.1	3	383,299–385,208	1302	1	433	48.63	9.26
*StCIPK06*	Soltu.DM.03G001400.1	3	1,308,274–3,10,351	1113	2	370	42.44	8.59
*StCIPK07*	Soltu.DM.04G031510.2	4	65,585,586–65,591,402	1344	14	447	50.63	6.38
*StCIPK08*	Soltu.DM.05G019250.1	5	43,311,668–43,315,613	1485	12	494	56.43	7.64
*StCIPK09*	Soltu.DM.05G022320.1	5	47,506,266–47,510,051	1338	1	445	50.20	9.07
*StCIPK10*	Soltu.DM.05G023210.1	5	48,492,654–48,497,748	1371	12	456	51.44	6.45
*StCIPK11*	Soltu.DM.06G002800.1	6	2,846,349–2,848,922	1341	2	446	50.96	8.47
*StCIPK12*	Soltu.DM.06G002810.1	6	2,851,745–2,855,743	1413	1	470	53.82	8.27
*StCIPK13*	Soltu.DM.06G010870.1	6	31,710,868–31,712,193	1326	1	441	49.74	9.10
*StCIPK14*	Soltu.DM.06G010880.1	6	31,745,058–31,746,792	1332	1	443	50.07	8.90
*StCIPK15*	Soltu.DM.06G024260.1	6	50,088,724–50,090,478	1353	1	450	51.02	8.92
*StCIPK16*	Soltu.DM.06G032750.1	6	57,566,917–57,568,428	1260	2	419	48.00	8.27
*StCIPK17*	Soltu.DM.07G000510.1	7	636,765–638,572	1275	1	424	47.66	9.08
*StCIPK18*	Soltu.DM.09G010080.1	7	18,714,464–18,716,381	1413	1	470	53.04	8.59
*StCIPK19*	Soltu.DM.08G015880.1	8	40,832,281–40,834,116	1371	1	456	52.35	8.63
*StCIPK20*	Soltu.DM.09G010290.1	9	14,851,369–14,853,146	1353	1	450	50.95	8.54
*StCIPK21*	Soltu.DM.09G025570.1	9	55,141,943–55,143,603	1317	2	437	49.44	8.90
*StCIPK22*	Soltu.DM.10G022340.1	10	53,624,978–53,626,957	1311	1	436	49.23	9.36
*StCIPK23*	Soltu.DM.11G018660.1	11	35,353,412–35,361,571	1398	13	465	53.07	7.24
*StCIPK24*	Soltu.DM.12G027440.1	12	2,229,648–2,31,821	1299	1	432	48.60	9.00
*StCIPK25*	Soltu.DM.12G026670.4	12	2,830,602–2,838,182	1341	14	446	50.63	8.87
*StCIPK26*	Soltu.DM.12G018730.1	12	18,210,942–18,212,405	1464	1	487	54.77	9.18
*StCIPK27*	Soltu.DM.12G002020.1	12	59,751,790–59,759,517	1596	11	531	60.01	5.97

^1^ Chr., chromosome; ^2^ CDS, coding sequence; ^3^ aa, amino acids; ^4^ MW, molecular mass; ^5^ pI, isoelectric point.

## Data Availability

Not applicable.

## Supplementary Materials

The following are available online at www.mdpi.com/article/10.3390/ijms222413535/s1.
