# Supplementary material for: Genome-Wide Identification, Characterization and Expression Analysis of the CIPK Gene Family in Potato (Solanum tuberosum L.) and the Role of StCIPK10 in Response to Drought and Osmotic Stress
Source: Int J Mol Sci. 2021 Dec 16;22(24):13535. doi: 10.3390/ijms222413535 (PMC8708990; doi:10.3390/ijms222413535)
Supplement: Supplementary file 1 [file ijms-22-13535-s001.zip › Table S1 Duplication information for the CIPK family in potato and Arabidopsis thaliana. .pdf]

Supplementary Table S1. The duplication information of *CIPK* family in potato and *arabidopsis thaliana*.

Locus1	Gene ID	Locus 2	Gene ID	Ka	Ks	Ka/ Ks	Million Years Ago (MYA)
AtCIPK14	AT5G01820	StCIPK22	Soltu.DM.10G022340.1	0.287456	2.80949	0.102316	93.65
AtCIPK14	AT5G01820	StCIPK16	Soltu.DM.06G032750.1	1.68266	1.36779	1.23021	45.59
AtCIPK04	AT4G14580	StCIPK05	Soltu.DM.03G000420.1	0.346209	3.72463	0.092951246	124.15
AtCIPK07	AT3G23000	StCIPK21	Soltu.DM.09G025570.1	1.86935	3.81655	0.489802	127.22
AtCIPK01	AT3G17510	StCIPK10	Soltu.DM.05G023210.1	1.16226	1.39516	0.833065	46.51
AtCIPK07	AT3G23000	StCIPK05	Soltu.DM.03G000420.1	0.358529	6.16715	0.0581353	205.57
AtCIPK22	AT2G38490	StCIPK22	Soltu.DM.10G022340.1	0.3859	2.8223	0.136732452	94.08
AtCIPK11	AT2G30360	StCIPK11	Soltu.DM.06G002800.1	0.3337	2.0949	0.159291613	69.83
AtCIPK11	AT2G30360	StCIPK16	Soltu.DM.06G032750.1	0.3004	2.5303	0.1187211	84.34
AtCIPK17	AT1G48260	StCIPK10	Soltu.DM.05G023210.1	0.233	4.3122	0.054032744	143.74
StCIPK01	Soltu.DM.01G005660.2	StCIPK23	Soltu.DM.11G018660.1	0.2917	0.8693	0.335557345	28.98
StCIPK06	Soltu.DM.03G001400.1	StCIPK04	Soltu.DM.02G015770.1	0.283	1.4994	0.188742164	49.98
StCIPK11	Soltu.DM.06G002800.1	StCIPK16	Soltu.DM.06G032750.1	0.2561	1.935	0.132351421	64.5
StCIPK27	Soltu.DM.12G002020.1	StCIPK10	Soltu.DM.05G023210.1	0.2182	1.053	0.207217474	35.1
StCIPK24	Soltu.DM.12G027440.1	StCIPK17	Soltu.DM.07G000510.1	0.1059	1.5604	0.067867214	52.01
StCIPK13	Soltu.DM.06G010870.1	StCIPK14	Soltu.DM.06G010880.1	0.0217395	0.0294931	0.737104611	0.98
