# Supplementary material for: Genome-Wide Identification, Characterization and Expression Analysis of the CIPK Gene Family in Potato (Solanum tuberosum L.) and the Role of StCIPK10 in Response to Drought and Osmotic Stress
Source: Int J Mol Sci. 2021 Dec 16;22(24):13535. doi: 10.3390/ijms222413535 (PMC8708990; doi:10.3390/ijms222413535)
Supplement: Supplementary file 1 [file ijms-22-13535-s001.zip › Table S2 Conserved motifs of 27 StCIPK genes in potato..pdf]

Table S2. The conserved motifs of 27 *StCIPK* genes in potato.

Motif Name	Motif logo	E-Value	Width	Site Count	possible motif
Motif 1	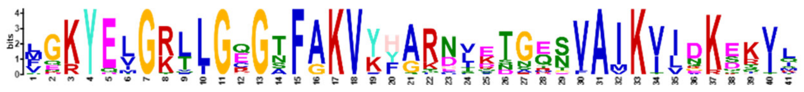	1.4e-544	41	26	MERYEVGRLLGQGTFAKVYYARNIKTGQSVAIKVIDKEKVL
Motif 2	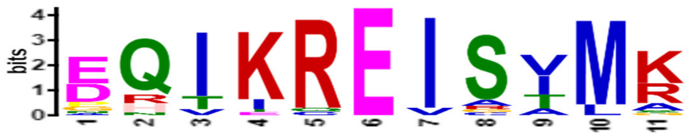	1.8e-121	11	26	EQIKREISVMR
Motif 3	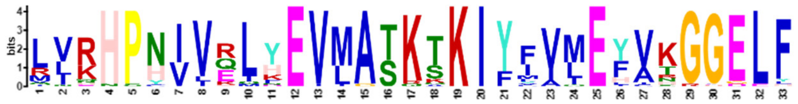	1.1e-526	33	26	LVRHPNIVHLYEVMATKTKIYFIMEYCKGGELF
Motif 4	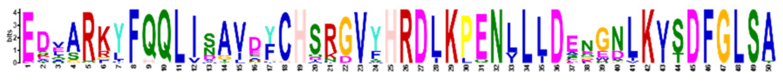	2.4e-994	50	27	EDVARKYFQQLISAVDFCHSRGVFHRDLKPENLLLDENENLKVSDFGLSA
Motif 5	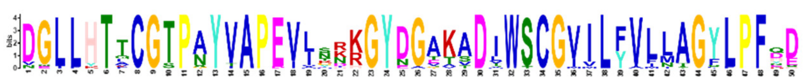	4.6e-1061	50	27	DGLLHTTCGTPAYVAPEVISRKGYDGAKADIWSCGVILFVLLAGYLPFQD
Motif 6	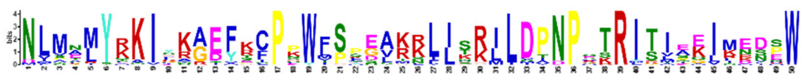	1.3e-703	50	27	NLMTMYRKIYKGEFRCPKWTSPELKRLLTRLLDINPVTRITIEEIKNDPW
Motif 7	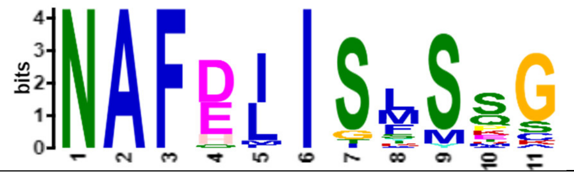	1.4e-116	11	25	NAFDIISMSSG
Motif 8	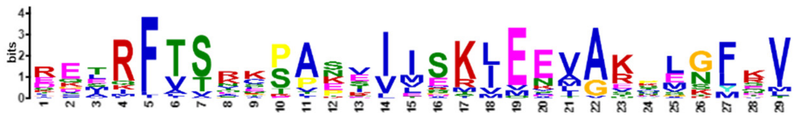	5.3e-197	29	27	RETRFTSKCPANEIISKIEEAAKPLGFDV

Motif 9	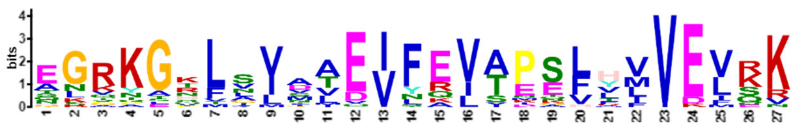	9.3e-174	27	25	AGRKGNLNVATEVFQVAPSLHM VEVRK
Motif 10	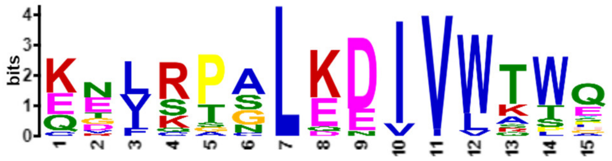	5.9e-080	15	18	EEVRPALKDIVWTWQ
