# Supplementary material for: Genome-Wide Identification, Characterization and Expression Analysis of the CIPK Gene Family in Potato (Solanum tuberosum L.) and the Role of StCIPK10 in Response to Drought and Osmotic Stress
Source: Int J Mol Sci. 2021 Dec 16;22(24):13535. doi: 10.3390/ijms222413535 (PMC8708990; doi:10.3390/ijms222413535)
Supplement: Supplementary file 1 [file ijms-22-13535-s001.zip › Table S3 Cis-elements in StCIPK promoter regions, including sequence and function..pdf]

Table S3. The information of *cis*-element in promoter region including sequence and function.

Respose of type	*Cis*-element	Sequence	Function of *cis* -element
Light responsive element	3-AF1 binding site	AAGAGATATTT	light responsive element
	AE-box	AGAAACAA	part of a module for light response
	Box 4	ATTAAT	part of a conserved DNA module involved in light responsiveness
	GA-motif	AAAGATGA	part of a light responsive element
		AAGGAAGA	part of a light responsive element
		ATAGATAA	part of a light responsive element
	TCT-motif	TCTTAC	part of a light responsive element
	Box I	TTTCAAA	light responsive element
	GT1-motif	GGTTAA	light responsive element
	Sp1	GGGCGG	light responsive element
		CC(G/A)CCC	light responsive element
	I-box	ATGATATGA	part of a light responsive element
	TCCC-motif	TCTCCCT	part of a light responsive element
	ACE	CTAACGTATT	cis-acting element involved in light responsiveness
		GACACGTATG	cis-acting element involved in light responsiveness
		ACGTGGA	cis-acting element involved in light responsiveness
		AAAACGTTTA	cis-acting element involved in light responsiveness
	G-box	TGACGTGG	cis-acting regulatory element involved in light responsiveness
		CACATGG	cis-acting regulatory element involved in light responsiveness
		TACGTG	cis-acting regulatory element involved in light responsiveness
		CCACGTAA	cis-acting regulatory element involved in light responsiveness
		CACGAC	cis-acting regulatory element involved in light responsiveness
		CACGTGG	cis-acting regulatory element involved in light responsiveness
		CACGTT	cis-acting regulatory element involved in light responsiveness
		CACACATGGAA	cis-acting regulatory element involved in light responsiveness
		CAGACGTGGCA	cis-acting regulatory element involved in light responsiveness
		CACGTT	cis-acting regulatory element involved in light responsiveness
		CACGTA	cis-acting regulatory element involved in light responsiveness
		GCCACGTGGA	cis-acting regulatory element involved in light responsiveness
		CACGTG	cis-acting regulatory element involved in light responsiveness
		CACGTC	cis-acting regulatory element involved in light responsiveness
	GATA-motif	GATAGGG	part of a light responsive element
		AAGGATAAGG	part of a light responsive element
		GATAGGA	part of a light responsive element
	box II	TCCACGTGGC	part of a light responsive element
	G-box	TAACACGTAG	cis-acting regulatory element involved in light responsiveness
		TAAACGTG	cis-acting regulatory element involved in light responsiveness
		CACGTG	cis-acting regulatory element involved in light responsiveness
		ACACGTGT	cis-acting regulatory element involved in light responsiveness
		CACATGG	cis-acting regulatory element involved in light responsiveness
	MRE	AACCTAA	MYB binding site involved in light responsiveness
	AT1-motif	ATTAATTTTACA	part of a light responsive module
	ATCT-motif	AATCTGATCG	part of a conserved DNA module involved in light responsiveness
	chs-CMA1a	TTACTTAA	part of a light responsive element
	LAMP-element	CTTTATCA	part of a light responsive element

	GAGA-motif	GATAGGG	part of a light responsive element
	sbp-CMA1c	CTTTATCTCTTCCA	part of a light responsive element
	GTGGC-motif	GATTCTGTGGC	part of a light responsive element
	CAG-motif	GAAAGGCAGAC	part of a light responsive element
Stress response			
	ARE	TGGTTT	cis-acting regulatory element essential for the anaerobic induction
	TC-rich repeats	GTTTTCTTAC	cis-acting element involved in defense and stress responsiveness
		ATTCTCTAAC	cis-acting element involved in defense and stress responsiveness
		ATTTTCTCCA	cis-acting element involved in defense and stress responsiveness
	MBS	CGGTCA	MYB Binding Site
		CAACTG	MYB binding site involved in drought-inducibility
		TAACTG	MYB binding site involved in drought-inducibility
	LTR	CCGAAA	cis-acting element involved in low-temperature responsiveness
	GC-motif	CCCCCG	enhancer-like element involved in anoxic specific inducibility
	MYB	CAACCA	cis-acting element involved in defense and stress responsiveness
	MYC	CATGTG	cis-acting element involved in the abscisic acid responsiveness
	WUN-motif	AAATTTCTT	cis-acting element involved in the abscisic acid responsiveness
	DRE-core	GCCGAC	cis-acting element involved in defense and stress responsiveness
Hormone response	O2-site	GATGATGTGG	cis-acting regulatory element involved in zein metabolism regulation
	CGTCA-motif	CGTCA	cis-acting regulatory element involved in the MeJA-responsiveness
	AuxRR-core	GGTCCAT	cis-acting regulatory element involved in auxin responsiveness
	P-box	CCTTTTG	gibberellin-responsive element
	TGACG-motif	TGACG	cis-acting regulatory element involved in the MeJA-responsiveness
	GARE-motif	TCTGTTG	gibberellin-responsive element
		AAACAGA	gibberellin-responsive element
	TATC-box	TATCCCA	cis-acting element involved in gibberellin-responsiveness
	TGA-box	TGACGTAA	auxin-responsive element
	CGTCA-motif	CGTCA	cis-acting regulatory element involved in the MeJA-responsiveness
	AT-rich sequence	TAAAATACT	element for maximal elicitor-mediated activation (2copies)
	ABRE	GCAACGTGTC	cis-acting element involved in the abscisic acid responsiveness
	TCA-element	TCAGAAGAGG	cis-acting element involved in salicylic acid responsiveness
		CCATCTTTTT	cis-acting element involved in salicylic acid responsiveness
		CAGAAAAGGA	cis-acting element involved in salicylic acid responsiveness
Growth related	CAT-box	GCCACT	cis-acting regulatory element related to meristem expression
	GCN4_motif	TGTGTCA	cis-regulatory element involved in endosperm expression
	HD-Zip 1	CAAT(A/T)ATTG	element involved in differentiation of the palisade mesophyll cells
	RY-element	CATGCATG	cis-acting regulatory element involved in seed-specific regulation
Circadian rhythm	MSA-like	(T/C)C(T/C)AACGG(T/C)(T/C)A	cis-acting element involved in cell cycle regulation
	circadian	CAAAGATATC	cis-acting regulatory element involved in circadian control
		CAANNNNATC	cis-acting regulatory element involved in circadian control
