# Supplementary material for: Genome-Wide Identification, Characterization and Expression Analysis of the CIPK Gene Family in Potato (Solanum tuberosum L.) and the Role of StCIPK10 in Response to Drought and Osmotic Stress
Source: Int J Mol Sci. 2021 Dec 16;22(24):13535. doi: 10.3390/ijms222413535 (PMC8708990; doi:10.3390/ijms222413535)
Supplement: Supplementary file 1 [file ijms-22-13535-s001.zip › Table S4 CBL gene family in potato.pdf]

Table S4. The information of CBL gene family in potato.

Gene	PGSC number	Chromosome	Location	Coding sequence (bp)
StCBL1	Sotub02g009050.1.1	2	16 010 030~160 10 704	675
StCBL2	Sotub03g006740.1.1	3	12 691 786~12 693 731	660
StCBL3	Sotub03g013280.1.1	3	45 441 374~45 439 308	645
StCBL4	Sotub06g016090.1.1	6	38 295 508~38 293 405	645
StCBL5	Sotub06g021020.1.1	6	44 283 937~44 286 430	648
StCBL6	Sotub07g028000.1.1	7	55 802 587~55 807 862	660
StCBL7	Sotub08g006040.1.1	8	1 065 129~1 068 732	648
StCBL8	Sotub08g009460.1.1	8	5 160 429~5 157 449	642
StCBL9	Sotub08g009430.1.1	8	5 203 153~5 200 751	642
StCBL10	Sotub08g014600.1.1	8	36 125 583~36 132 372	774
StCBL11	Sotub08g023240.1.1	8	50 845 078~50 839 971	642
StCBL12	Sotub10g005740.1.1	10	893 952~897 316	669
StCBL13	Sotub12g013550.1.1	12	10 195 735~10 202 211	675
