# Supplementary material for: Genome-Wide Identification, Characterization and Expression Analysis of the CIPK Gene Family in Potato (Solanum tuberosum L.) and the Role of StCIPK10 in Response to Drought and Osmotic Stress
Source: Int J Mol Sci. 2021 Dec 16;22(24):13535. doi: 10.3390/ijms222413535 (PMC8708990; doi:10.3390/ijms222413535)
Supplement: Supplementary file 1 [file ijms-22-13535-s001.zip › Table S5. Sequences of the primers used in this study.pdf]

Table S5. Sequences of the primers used in this study.

Gene	Primer sequence	
	Forward	Reverse
Primers for real-time quantitative PCR		
StCIPK01	AGCCTCTCGGTTTTGACGTT	CCGCACCTCGACCATATGAA
StCIPK02	GCGCATCACAATTACCGAGGC	GCATTCATAGTCAGCGGTGC
StCIPK03	GGCTTCTGTTGGGCAGGTAT	TCGCTACGCTCTCATTCGTC
StCIPK04	CGCTGCAGTCGATTTTTGTCA	CACACGTTGTGTGGAGGAGA
StCIPK05	CCACGGCCTTTGTTTAGACG	ATTAAGACGGGACATGGCGG
StCIPK06	AGTCCACGCCCACTATTGTC	CTTGCATTTTCACCGTCCC
StCIPK07	TGGACTTAGCGCATCACCTG	GGATGACACCACAGGACCAG
StCIPK08	ACCCGAATCCACTCACAAGG	ATCTCATTAGCAGGCGACCG
StCIPK09	GCGAAGCCTGCCTCTTCTAT	CCCTCACCCGATCCCTCTAA
StCIPK10	ACTTTGGGCTCAGTGCGTTA	CGCGCCATCATATCCTCTGT
StCIPK11	TACGACACCCACACATCGTC	TTTGCGAACAATTCGCCTCC
StCIPK12	AAGGCTAAAAGAGGACGCCG	CCCGGTGATAGACACCCCTA
StCIPK13	AGCGGCAAGATGGGTTACTC	CACCCCACAAGACCAGATGT
StCIPK14	AAGGTGGCGAGCTTTTCAAC	AAACATCTCGGCTGTGGCAA
StCIPK15	CGAAATGCTCTGCCTCAACG	TCGTCACTTCTGGTGCAACT
StCIPK16	CCACCGCGATTTGAAACCTG	CATACGCCGGAGATCCACAA
StCIPK17	CGAAGATCGCGAAAGGCAAG	CCGTAAATGCACTTAGCCCG
StCIPK18	CACCGAGACCTAAAACCGGA	CACCGTCATAGCCTCTCCTG
StCIPK19	GCGTATTTCGAGTTCCCTCCT	TTGGATTGAAAACGCCCGTG
StCIPK20	ACTCAAAGGTGGGCTAACGG	AGCGAATTCCAGGACGAAGT
StCIPK21	CGGTGAGCTTTTCACGAAGC	CCGTTTTGGTGGCAGAAGTG
StCIPK22	TGCTAGGGATGTGAGGACGA	GAAGGCGCACGATATGAGGA
StCIPK23	AGCCCCTCGGTTTTGATGTT	ATCTCCTTTTGCCTTCCGCA
StCIPK24	AAGGTAACCGATTTCGGGCT	CCAAGTACTTCAGGGGCAACA
StCIPK25	TTTCCCGCCAACCTGCTAAA	TGCCGTTACCCCCTCAATTC
StCIPK26	TTGTCCGCCACCCTAACATT	ACGTCTTCTTTGAGCCTCCC
StCIPK27	TGCGAGTAGGGAAATACGAGC	GTCCTGGATCCGATTCTTCTCC
StEf1a	CAAGGATGACCCAGCCAAG	TTCCTTACCTGAACGCCTGT
AmiRNA-StCIPK10	GCGTGTGACAGTAACTAACGGCAT	Sequence from TIANGEN BIOTECH(BEIJING) CO., LTD. (not publish)
St18S RNA	TTAGAGGAAGGAGAAGTCGTAACAA	Sequence from TIANGEN BIOTECH(BEIJING) CO., LTD. (not publish)
StABI1	GACTAGACCCGTGGCAGTTC	CAGTTGCGAATGCGTCTTCC
StABI3	AGGGCCAATACAGGGGTACA	AAATTGCGACGGAGACCAGG
StCOR47	AGGAGAAATTGCCAGGTGGAG	TTCCTCTTCAGTCTTTGAGTGGT
StKIN1	TGAGCTACCAAGCTGGTCAAG	TTTTAACTGCATCTGCCGCTC
StRD22	ACAATTACGCGGCGAAAGAC	TTCCGGCATCTTCTCTGAGC

StRD29B	CCAACAATGAGGAGGCAGGT	TGTAGGCGATTCTTGCGGAG
Primers for overexpression vector		
StCIPK10-OE	CGGGGGACGAGCTCGGTACCATGGTGTTGGTACAACAGGAA	CTTCGTCGACTCTAGACTAGCTATCACAACAAACAGTAGG
Primers for amiRNA vector		
I miR-s	gaTGTGACAGTAACTAACGGCATtctctcttttgtattcc	
II miR-a	gaATGCCGTTAGTTACTGTCACAtcaaagagaatcaatga	
III miR*s	gaATACCGTTAGTTAGTGTCACTtcacaggtcgtgatatg	
IV miR*a	gaAGTGACACTAACTAACGGTATtctacatatatattcct	
A	CTGCAAGGCGATTAAGTTGGGTAAC	
B	GCGGATAACAATTTCACACAGGAAACAG	
Primers for subcellular localization		
StCIPK10-EGFP	CGGGGGACGAGCTCGGTACCATGGTGTTGGTACAACAGGAA	CCATGTCGACTCTAGAGCTATCACAACAAACAGTAGGTAGG
Primers for identifying transgenic plants		
HPT	GTGATTTCATATGCGCGATTGCTG	ACGAGTGCTGGGGCGTCGGTTTCC
NPT-II	GCTATGACTGGGCACAACAG	ATACCGTAAAGCACGAGGAA
Primers for 5′RACE		
StCIPK10-Inner	CGCGGATCCGAACACTGCGTTTGCTGGCTTTGATG	CCACATGACCAGGTATCAGATGCC
StCIPK10-Outer	GCTGATGGCGATGAATGAACACTG	CTCTTGCATCACTTTCAACCTTCCG
Primers for yeast one-hybrid assays		
pGBKT7-StCIPK10	AGGAGGACCTGCATATGATGGTGTTGGTACAACAGGAAGAC	TAGTTATGCGGCCGCTGCAGCTAGCTATCACAACAAACAGTAGG
pGADT7-StCBL1	GAAAGGTCGAATTGGGTACCATGTTGTCGTGCTTAGGTTCTTACC	CGAGCTCGATGGATCCTCAATCCCAAATCAGGTCGTCC
pGADT7-StCBL2	GAAAGGTCGAATTGGGTACCATGCATTCTC	CGAGCTCGATGGATCCTCAGTTTCCATATACCAATTGTGAG
pGADT7-StCBL3	GAAAGGTCGAATTGGGTACCATGGGCTGCTTTCACTCAAAAATC	CGAGCTCGATGGATCCCTAGACTTCCGAATCTTCCACCTC
pGADT7-StCBL4	GAAAGGTCGAATTGGGTACCATGGGCTGCTTTCACTCAAAAA	CGAGCTCGATGGATCCCTAAATTTCTGAATCATCAACCTCA
pGADT7-StCBL5	GAAAGGTCGAATTGGGTACCATGGGCTGTTTTCATTCTACAG	CGAGCTCGATGGATCCTCATAGTTGGGTAGCAGCTTC
pGADT7-StCBL6	GAAAGGTCGAATTGGGTACCATGCTGCAGTTCTTAGGTTC	CGAGCTCGATGGATCCTTAGGTGTCCTCAACTCTTGAATG

pGADT7-StCBL7	GAAAGGTCGAATTGGGTACCATGGGCTGTTTTAGCTCTAAGGTGG	CGAGCTCGATGGATCCTCAAGTAGCTCCTTCATCAACTTC
pGADT7-StCBL8	GAAAGGTCGAATTGGGTACCATGGGGTGTGCTTTAAGGA	CGAGCTCGATGGATCCTCAGAAATCCTTGTAAATCTCATCA
pGADT7-StCBL9	GAAAGGTCGAATTGGGTACCATGGGTTGTGCTATAAGGAAAC	CGAGCTCGATGGATCCTTACTTGAGATATGGAATTGTCAT
pGADT7-StCBL10	GAAAGGTCGAATTGGGTACCATGGATTCCACGCGAAGTTCTC	CGAGCTCGATGGATCCTCACAACAAATGGGTTTTCTCCG
pGADT7-StCBL11	GAAAGGTCGAATTGGGTACCATGGGCTGCTTTAATTCTAAGGTGA	CGAGCTCGATGGATCCTTATGTAGCAACTTCATCAACTTCA
pGADT7-StCBL12	GAAAGGTCGAATTGGGTACCATGTCGTATTGCTTTGAGGGG	CGAGCTCGATGGATCCTCAAGTATCCGGAACTCTTGAGTG
pGADT7-StCBL13	GAAAGGTCGAATTGGGTACCATGTTGCAGTGCCTAGAGGGG	CGAGCTCGATGGATCCTCAGGTATCCTCAACTCTGGAATG
Primers for BIFC		
pSPYCE-35S-StCIPK10	CACGGGGGACTCTAGAATGGTGTTGGTACAACAGGAAGA	TACATCCCGGGAGCGGTACCGCTATCACAACAAACAGTAGGT
pSPYNE-35S-StCBL11	CACGGGGGACTCTAGAATGGGCTGCTTTAATTCTAAGGTG	TCCATCCCGGGAGCGGTACCTGTAGCAACTTCATCAACTTCAG
