# Supplementary figures and images for: Genome-Wide Identification, Characterization and Expression Analysis of the CIPK Gene Family in Potato (Solanum tuberosum L.) and the Role of StCIPK10 in Response to Drought and Osmotic Stress
Source: Int J Mol Sci. 2021 Dec 16;22(24):13535. doi: 10.3390/ijms222413535 (PMC8708990; doi:10.3390/ijms222413535)

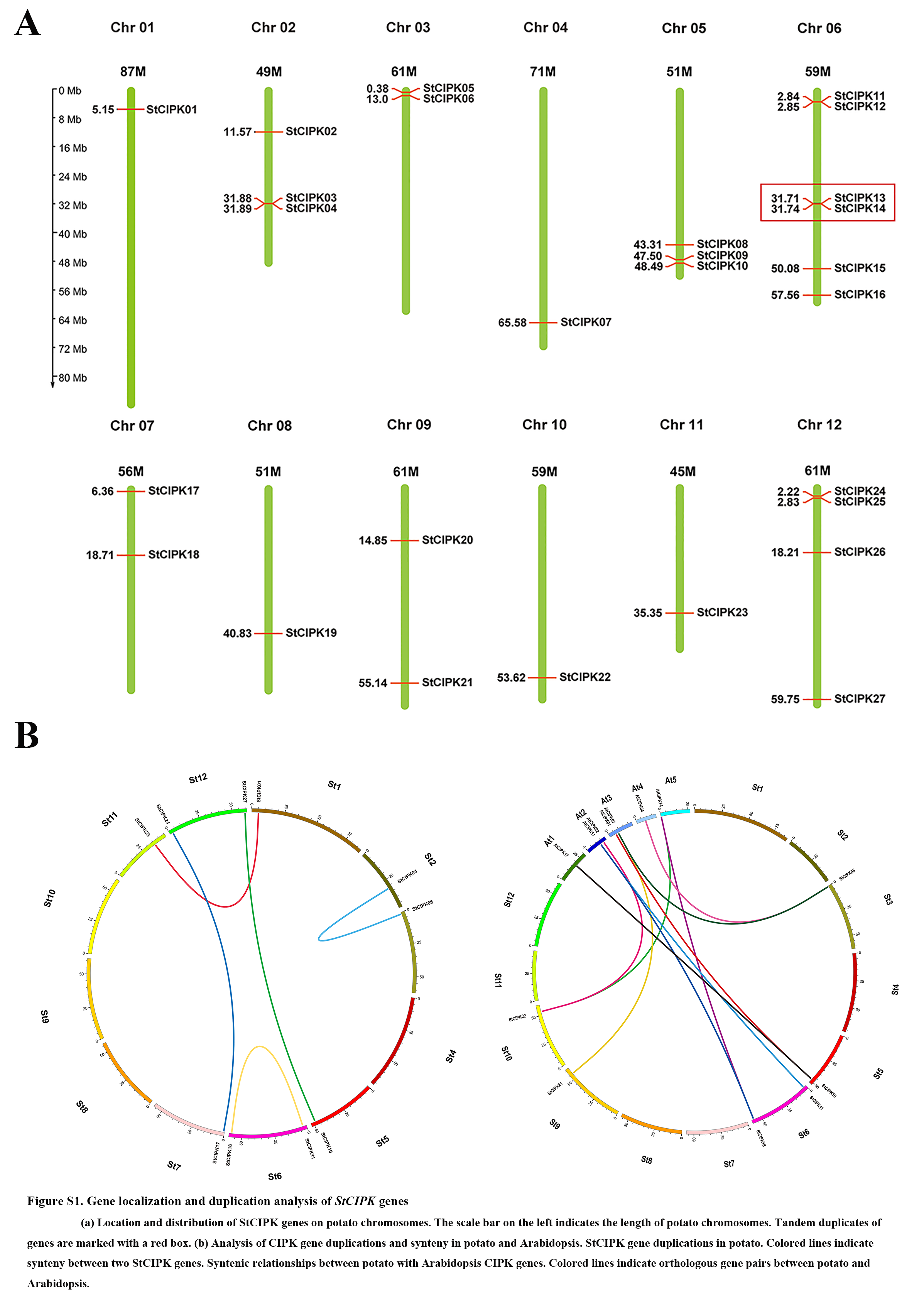

Supplement: Supplementary file 1 [file ijms-22-13535-s001.zip › Figure S1 Gene localization and duplication analysis of StCIPK genes.tif]

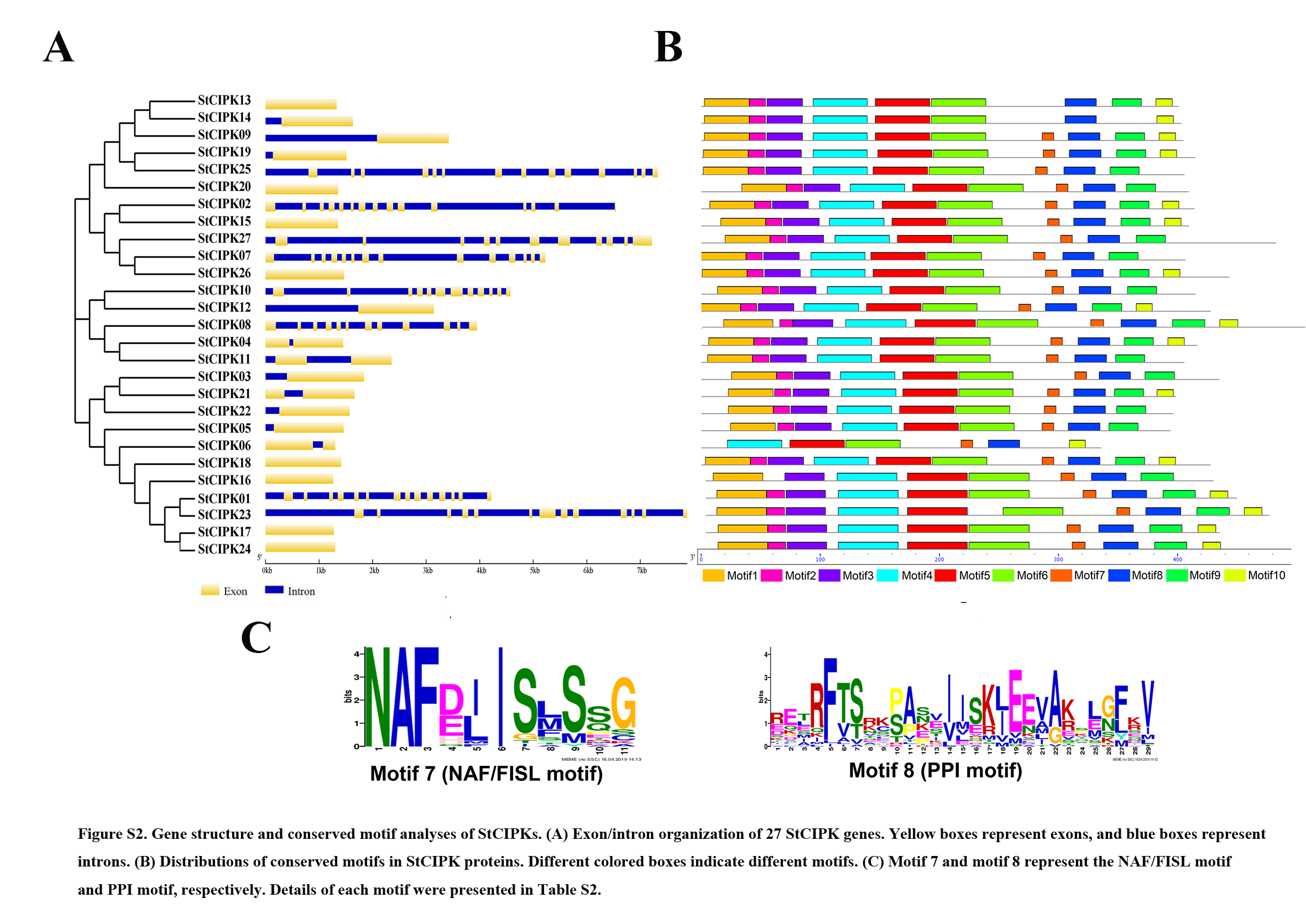

Supplement: Supplementary file 1 [file ijms-22-13535-s001.zip › Figure S2 Gene structure and conserved motif analyses of StCIPKs..tif]

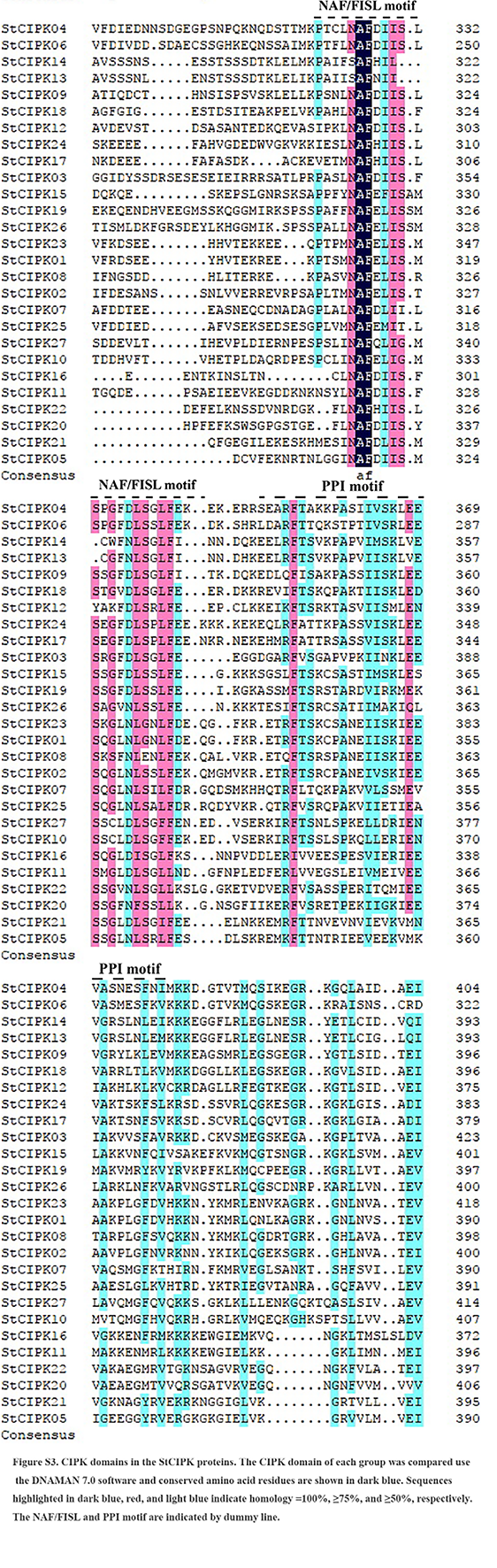

Supplement: Supplementary file 1 [file ijms-22-13535-s001.zip › Figure S3 CIPK domains in the StCIPK proteins.tif]

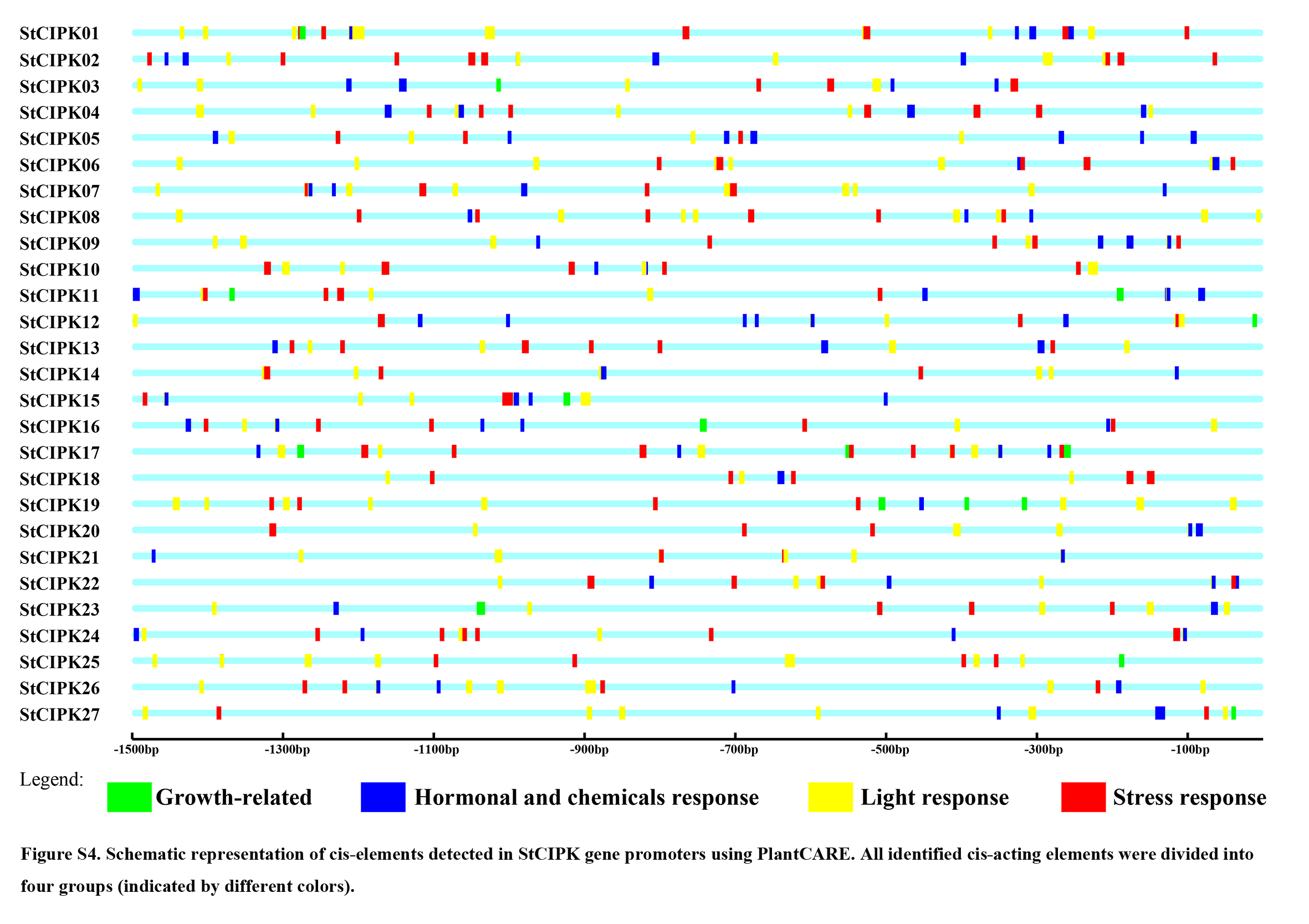

Supplement: Supplementary file 1 [file ijms-22-13535-s001.zip › Figure S4 Schematic representation of cis-elements detected in StCIPK gene promoters using PlantCARE..tif]
